# Signal peptide and N-glycosylation of N-terminal-CD2v determine the hemadsorption of African swine fever virus

**DOI:** 10.1128/jvi.01030-23

**Published:** 2023-09-28

**Authors:** Daniel Pérez-Núñez, Raquel García-Belmonte, Elena Riera, Marta H. Fernández-Sesma, Gonzalo Vigara-Astillero, Yolanda Revilla

**Affiliations:** 1 Microbes in Health and Welfare Department, Centro de Biología Molecular Severo Ochoa, CSIC-UAM, Madrid, Spain; Northwestern University Feinberg School of Medicine, Chicago, Illinois, USA

**Keywords:** ASFV, hemoadsorption, CD2v, N-glycosylation, signal peptide, NH/P68, Arm/07/CBM/c2

## Abstract

African swine fever virus (ASFV) is the cause of African swine fever (ASF), a devastating disease that affects domestic pigs and wild boar and is currently responsible for the largest animal epidemic. One of the characteristics of ASFV infection, but absent in naturally attenuated strains, is hemadsorption (HAD), a phenomenon that has been linked to virulence. In this study, we have shown that ASFV HAD depends exclusively on the Nt domain of the ASFV CD2v protein during infection. CD2v is a highly glycosylated protein, and we found that glycosylation is essential for HAD. However, despite the higher degree of CD2v glycosylation, only simultaneous N-glycosylation of two Asp residues on the Nt region is the determinant for HAD. On the contrary, we have demonstrated that the presence of a specific signal peptide sequence on CD2v not only influences the degree of CD2v glycosylation but is also critical for HAD, although not for CD2v localization. Finally, we have shown that the CD2v expression during infection of the non-HAD NH/P68 strain is not sufficient for HAD, despite its glycosylation and cell surface localization. Complementation studies of CD2v from NH/P68 with CD2v signal peptides from HAD+ strains indicate (i) that different signal peptides from different genotypes are able to restore HAD and (ii) the existence of a HAD-inhibitory sequence in NH/P68-CD2v. This study lays the molecular basis for ASFV HAD, which could be key for the study of virulent and pathogenic aspects of the virus, as well as the rational development of new vaccines against ASFV.

**IMPORTANCE:**

African swine fever virus (ASFV) is the cause of the current major animal epidemic worldwide. This disease affects domestic pigs and wild boars, has spread since 2007 through Russia, Eastern Europe, and more recently to Western European countries, and since 2018 emerged in China, from where it spread throughout Southeast Asia. Recently, outbreaks have appeared in the Caribbean, threatening the Americas. It is estimated that more than 900,000 animals have died directly or indirectly from ASFV since 2021 alone. One of the features of ASFV infection is hemoadsorption (HAD), which has been linked to virulence, although the molecular and pathological basis of this hypothesis remains largely unknown. In this study, we have analyzed and identified the key players responsible of HAD, contributing to the identification of new determinants of ASFV virulence, the understanding of ASFV pathogenesis, and the rational development of new vaccines.

## INTRODUCTION

African swine fever virus (ASFV), member of Asfaviridae family, is a complex, dsDNA virus, which infects domestic pigs and wild boar, causing African swine fever (ASF), a severe disease with up to 100% mortality (1, 2). ASFV was identified in Africa in 1921 where it is still endemic, displaying a first spread of genotype I strains from the continent, through Europe, between the 1960s and the 1990s. In 2007, outbreaks of genotype II strains were detected in the Caucasus from which the virus continued to spread through Russia, and then East Europe, affecting many countries (i.e., Romania, Poland, Latvia, and Hungary) and more recently reaching Western European countries, such as Belgium, Italy, and Germany (3). ASFV is widespread in Asia, since the detection of the first outbreaks in China in 2018 (4) affecting many Southeast Asian countries, including the Philippines, the Koreas, Vietnam or Indonesia, and very recently India (5 – 8). In 2021, ASFV reached Caribbean (9), now threatening other countries in the Americas. The epidemic caused by ASFV is currently out of control. Since January 2021, ASF has been reported to be present in five different world regions in 40 countries, affecting millions of pigs, more than 23,000 wild boars, and more than 990,000 animal losses (10). The development of novel, safe, and efficacious vaccines is urgently required to address the threat to worldwide pork production. To achieve this, one of the key aspects is the understanding of the molecular mechanisms of ASFV virulence.

The virulence of different ASFV strains varies considerably, from up to 100% mortality, in the case of currently circulating virulent genotype II strains, to attenuated strains that cause chronic disease without producing death. It has been speculated that one of the reasons for the different degrees of virulence is the ability of the virus to control the host immune response, in particular the innate immune response through the control of the cGAS/STING and JAK/STAT pathways, as we have previously described (11, 12). Additionally, other important aspect of ASFV biology that may play a role in virulence is the hemadsorption (HAD). HAD is the ability of the virus, and of the virus-infected cells, to bind erythrocytes (13). HAD is a characteristic that has been used for ASFV titration (14) and to phenotype ASFV strains to serogroups on the basis of antibody-mediated hemadsorption inhibition (HAI) (15), which has been suggested to be important in the cross-protection to be considered in vaccine design (16, 17). It is unclear what role HAD should play in the pathogenesis of ASF, although a role in dissemination and/or evasion of immune control has been speculated (18). What is clear is that naturally attenuated strains, both genotype I (19 – 21) and genotype II (22), lose their ability to hemadsorb, and thus, it is tempting to speculate that HAD could be one of the viral components involved in ASFV virulence (23). Moreover, interestingly, the virulence of certain strains of *Plasmodium falciparum* (the cause of malaria) has also been related to the hemadsorbing capacity of this pathogen (24). In particular, HAD in ASFV has been linked to at least two viral proteins, namely, EP402R, which encodes for the CD2v protein (25 – 27), and EP153R (28). In fact, the deletion of CD2v has itself caused the attenuation of certain virulent strains (29, 30), connecting CD2v-HAD and virulence, yet not in others (25, 31), and so, there is some controversy as to whether other viral genotype-dependent viral factors can modulate virulence. In this regard, the ASFV protein EP153R has been implicated both in HAD and in some degree of attenuation and/or protection when deleted from the viral genome together with other viral genes (32 – 35) and was described to be a needed factor, together with CD2v, for HAI-based serological grouping (17).

In this work, we have shown that CD2v on its own, and not EP153R, is responsible for the hemadsorbing phenotype, ruling out the role of EP153R previously proposed. In addition, we have further elaborated on the molecular determinants of CD2v in HAD, which depend exclusively on the N-terminal (Nt) domain of CD2v, both *in vitro* and during ASFV infection. Furthermore, we demonstrate the importance of N-glycosylation of this CD2v-Nt domain for HAD, which relies on two particular Asp (N) residues located within the CD2v-Nt domain. The impairment of simultaneous glycosylation of these two domains prevents HAD, despite glycosylation in other residues of the protein. In addition to glycosylation, the integrity of the CD2v-signal peptide, which may not be directly related to glycosylation, appears to be essential. Finally, and for the first time, we show that during the non-HAD NH/P68 strain infection, CD2v is expressed, a concept largely ignored. However, and importantly, CD2v-protein expression of NH/P68, despite being glycosylated and localized in the cell surface, is unable to induce HAD. Here, we demonstrate that this is not only because NH/P68 CD2v lacks any signal peptide but also because we have identified a HAD inhibitory sequence at the Nt end of CD2v of NH/P68. This inhibitory sequence is also able to prevent HAD in CD2v of HAD+ strains. Its deletion, together with the addition of the signal peptide from CD2v proteins of HAD strains, restores HAD capacity to CD2v of NH/P68.

## RESULTS

### Rosette formation requires CD2v but not EP153R expression

**Fig 1:**
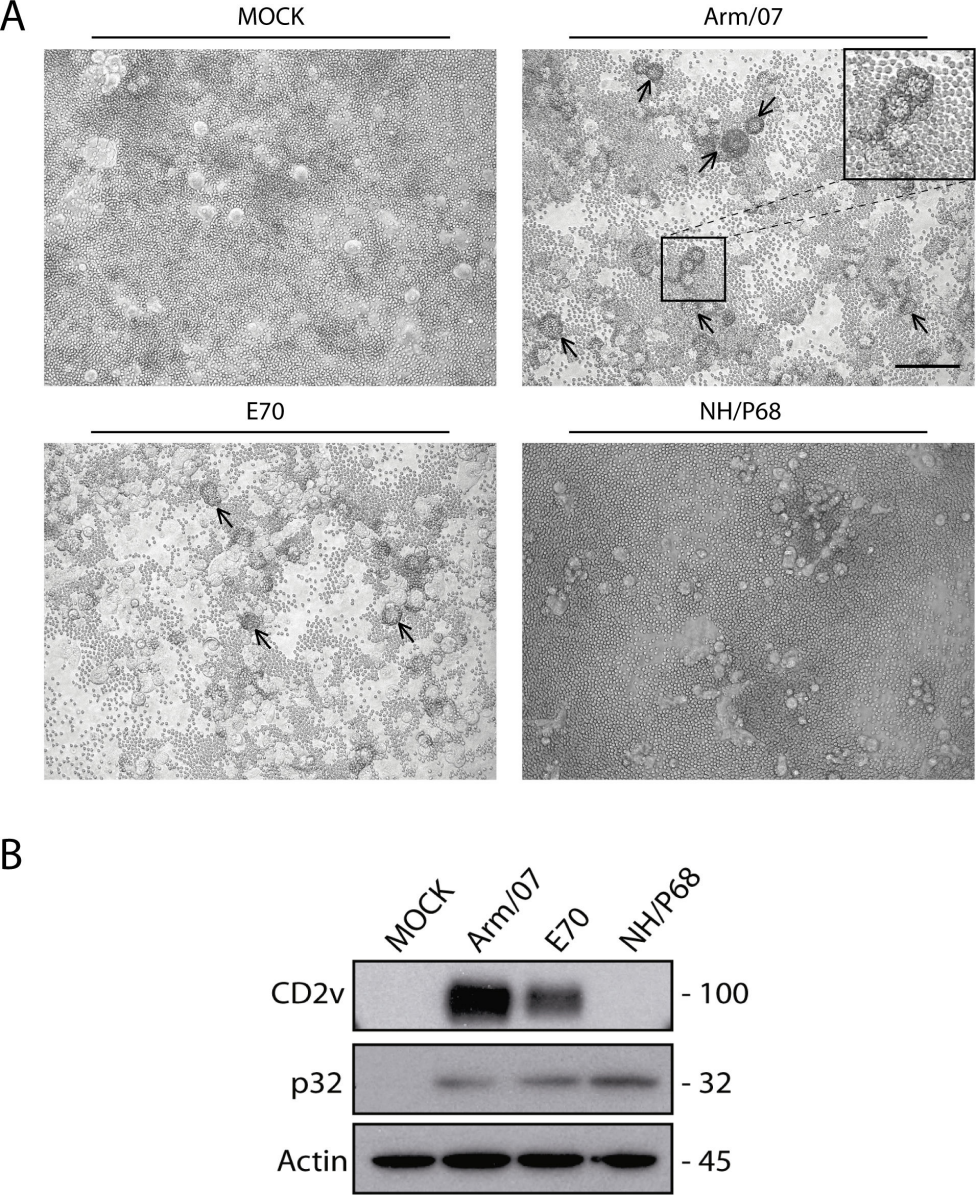
ASFV-infected COS-1 cells and porcine erythrocytes formed rosettes. COS-1 cells were MOCK-infected or infected with Arm/07/CBM/c2 (Arm/07), E70, or NH/P68 for 16 hpi (MOI = 1). Samples were then (**A**) incubated with porcine erythrocytes for further 24 h before observation under light microscope and (**B**) lysed in RIPA buffer, separated by 10% SDS-PAGE, and followed by immunoblotting with anti-ASFV CD2v, anti-ASFV p32, and anti-actin antibodies. Arrows indicate rosettes. Scale bar 100 µm.

CD2v (EP402R) and EP153R are ASFV proteins that were previously described to be involved in ASFV rosette formation and HAD (26 – 28), a phenomenon suggested to be linked to virulence (21, 22, 24, 29, 30). Toward the understanding of the molecular mechanisms mediating ASFV-HAD, we first set up an *in vitro* assay to analyze rosette formation in COS-1 cells. COS-1 cells were infected with different HAD+ ASFV isolates, such as the genotype II strain Arm/07/CBM/c2 (36) or the genotype I strain E70, or the non-HAD strain NH/P68 (21). After 16 hpi, porcine erythrocytes were added to the medium and incubated for further 24 h, and cells were observed for rosette formation. As expected, Fig. 1A shows rosettes in Arm/07/CBM/c2 and E70-infected cells when erythrocytes were added, while no rosettes were observed in NH/P68-infected cells. The expression of CD2v protein was detected with an anti-CD2v antibody generated in-house (37) in both Arm/07/CBM/c2 and E70-infected COS-1 cells but not in NH/P68 COS-1-infected cells (Fig. 1B). These results demonstrated the suitability of COS-1 cells and porcine erythrocytes for *in vitro* HAD assay.

**Fig 2:**
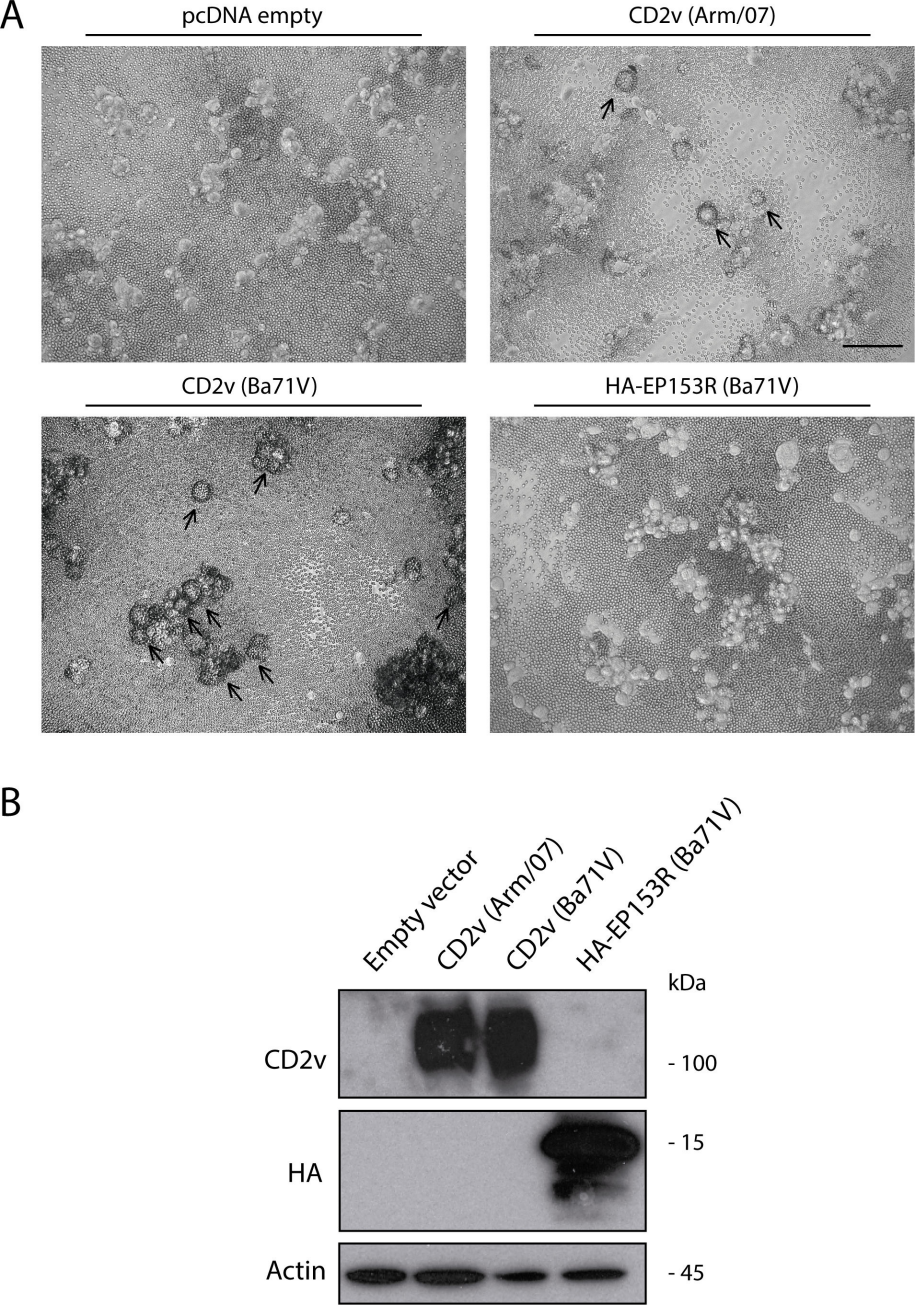
Rosette formation is mediated by CD2v and not by EP153R. COS-1 cells were transfected with empty vector or expression vectors for CD2v of Arm/07/CBM/c2 (Arm/07) or Ba71V strains or for HA-EP153R of Ba71V strain (1 µg/10^6^ cells). At 6 hpt, cells were infected with VV-T7 (MOI = 0.5) for 16 h and (**A**) incubated with porcine erythrocytes for further 24 h before observation under light microscope; and (**B**) they were lysed in RIPA buffer, separated by 10% SDS-PAGE, and followed by immunoblotting with anti-ASFV CD2v, anti-HA, and anti-actin antibodies. Arrows indicate rosettes. The empty vector pcDNA control image is used in Fig. 2, 3, and 5 because they are all part of the same internally controlled experiment. A representative image (*n* = 3) is shown. Scale bar 100 µm.

In order to study the individual contribution of each of the viral proteins suggested to be involved in HAD, we generated expression vectors of CD2v from HAD+ genotype II Arm/07/CBM/c2 strain and from the genotype I Ba71V strain, as well as HA-EP153R or empty vector, which were transfected in COS-1 cells and then infected with VV-T7, in order to increase the amount of the ectopically expressed proteins, as it was previously accomplished for other ASFV proteins (38); 24 h post-transfection (hpt), porcine erythrocytes cells were added to the medium, and the formation of rosettes was observed 24 h later, as explained above. As shown in Fig. 2, the expression of CD2v, from either Arm/07/CBM/c2 or Ba71V strains, was enough to induce rosette formation, whereas the individual expression of EP153R did not. Importantly, these results indicate that the expression of CD2v is necessary and sufficient for HAD formation, independently of the expression of any other ASFV protein, ruling out a role of EP153R in HAD.

### HAD directly depends on CD2v-Nt domain during ASFV infection

**Fig 3:**
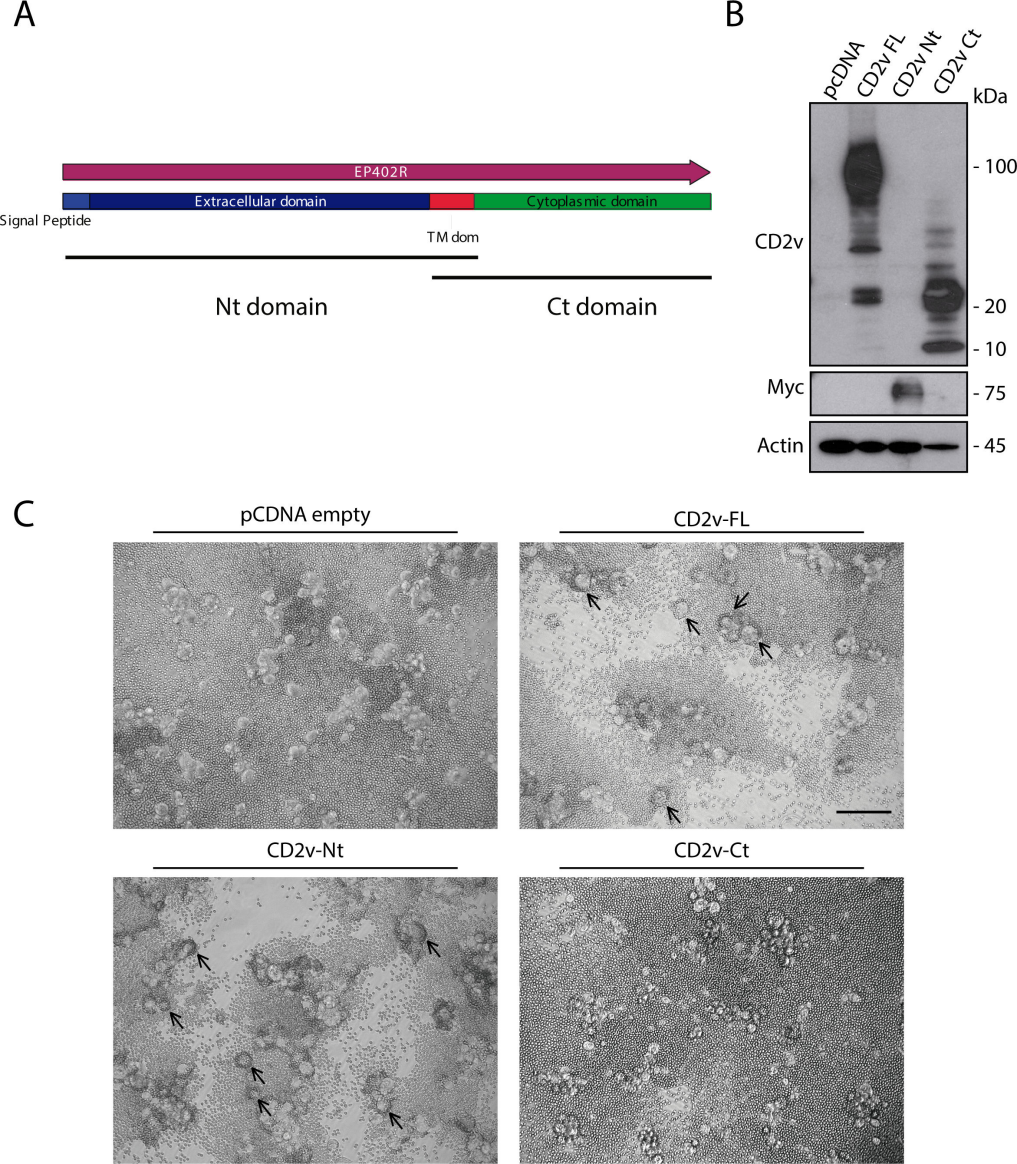
Ectopic expression of CD2v-Nt is sufficient and necessary for HAD. (**A**) Diagram of the Nt domain and Ct domain structure of CD2v. COS-1 cells were transfected (1 µg/ 10^6^ cells) with empty vector (pcDNA) or expression vectors for CD2v full-length (CD2v-FL), Nt domain (CD2v-Nt), and Ct domain (CD2v-Ct) of Arm/07/CBM/c2. At 6 hpt, cells were infected with VV-T7 (MOI = 0.5); samples were then (**B**) lysed in RIPA buffer, separated by 10% SDS-PAGE, and followed by immunoblotting with anti-ASFV CD2v, anti-myc, and anti-actin antibodies and (**C**) incubated with porcine erythrocytes for further 24 h before observation under light microscope. Arrows indicate rosettes. The empty vector pcDNA control image is used in Fig. 2, 3 and 5 because they are all part of the same internally controlled experiment. A representative image (*n* = 3) is shown. Scale bar 100 µm.

CD2v is a transmembrane protein displaying an extracellular Nt domain that contains a signal peptide and a cytoplasmic Ct domain. Aiming to identify the specific domain(s) involved in HAD, we generated two new vectors containing either the Nt domain or the Ct domain of CD2v of Arm/07/CBM/c2 together with the transmembrane domain in both cases (CD2v-Nt or CDv-Ct, respectively), as indicated in Fig. 3A. The ectopic expression of these vectors, together with CD2v full length (CD2v-FL), revealed that our anti-CD2v antibody specifically recognizes the Ct domain of CD2v, and hence, we have added myc tag to detect the CD2v-Nt vector (into its Ct end) (Fig. 3B).

As shown in Fig. 3C, the expression of either CD2v-Nt or CD2v-FL was sufficient to induce rosette formation in transfected COS-1 cells in the presence of erythrocytes. However, cells transfected with CD2v-Ct cannot induce the rosette formation. These results indicate that the ectopic expression of CD2v-Nt is necessary and sufficient for HAD, whereas CD2-Ct has no role on it.

**Fig 4:**
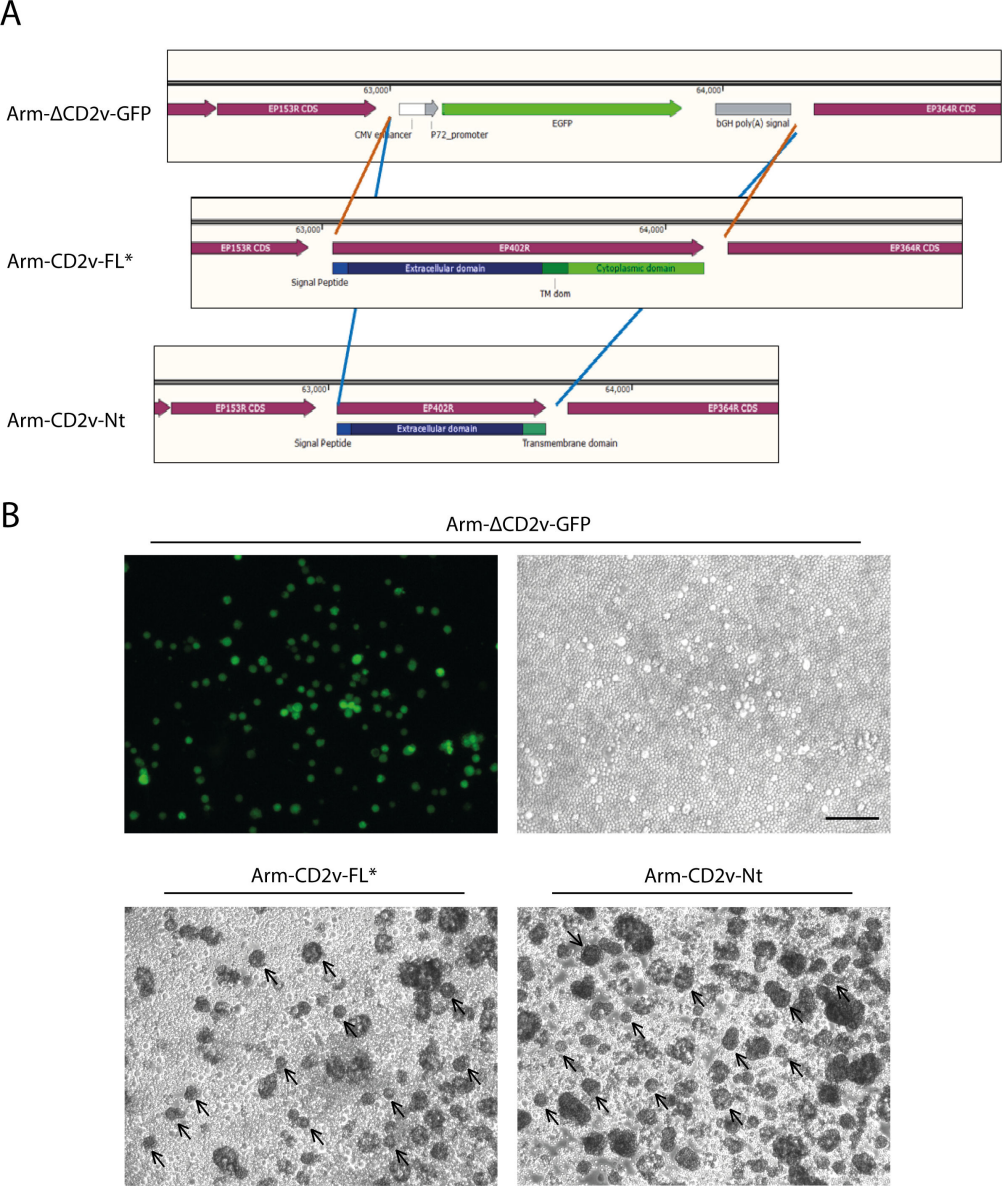
CD2v-FL and CD2v-Nt recover HAD ability from a non-HAD Arm-ΔCD2v-GFP recombinant virus during PAM infection. (**A**) Diagram of the recombinant viruses: Arm-ΔCD2v-GFP was used as a backbone, replacing GFP by CD2v-FL or CD2v-Nt to generate Arm-CD2vFL* and Arm-CD2v-Nt, respectively. (**B**) PAM cells were infected with Arm-ΔCD2v-GFP, Arm-CD2vFL*, or Arm-CD2v-Nt (MOI = 1). After 16 h, porcine erythrocytes were added to the medium, and 24 h later, cells were observed under fluorescent/light microscope to identify rosettes. Arrows indicate rosettes.

To confirm the direct involvement of CD2v-Nt in HAD during ASFV infection, we generated two recombinant viruses to analyze their ability to induce rosette formation by using a previously generated deletion mutant from Arm/07/CBM/c4 (36) lacking the EP402R gene (Arm-ΔCD2v-GFP), as a backbone to replace the EGFP gene by either full length CD2v (Arm-CD2v-FL*) or CD2v-Nt (Arm-CD2v-Nt), as indicated in Fig. 4A.

The generation of these recombinant viruses and the absence of parental virus contamination were first verified by PCR (Fig. S1) and then by Illumina sequencing and variant calling, which revealed only two SNPs between each of the Arm-CD2vNt and Arm-CD2vFL* recombinants and their parental Arm-ΔCD2v-GFP, indicating the almost absence of off-target mutations (Table S1). These data indicate that the difference between the three recombinants lies exclusively in CD2v: either the absence of CD2v (Arm-ΔCD2v-GFP) or the presence of the full-length protein (Arm-CD2v-FL*) or the Nt domain (Arm-CD2v-Nt). These recombinant viruses were used then to infect porcine alveolar macrophages (PAM) at a MOI = 1 for 16 h. At that time, we added porcine erythrocytes and incubated for further 24 h, before observation at both fluorescent and light microscopes. As it can be seen in Fig. 4B, Arm-ΔCD2v-GFP infection did not generate any rosette in the presence of erythrocytes, as expected. However, rosettes were observed in Arm-CD2v-FL*-infected PAM, indicating that CD2v-FL efficiently recovered the HAD phenotype. More importantly, Arm-CD2v-Nt-infected PAM induced rosettes in the presence of porcine erythrocytes.

It is important to note that the percentage of rosettes we observed in PAMs infected with the recombinant viruses is higher than that observed in transfected COS-1 (Table S2 and S3). This may be due to the difference between transfection efficiency vs. infection, but also to different cellular factors that may play a role in HAD. Accordingly, a similar percentage of rosettes is observed in infected COS-1 as in transfected COS-1 (Table S2).

Altogether these results clearly demonstrate that CD2v-Nt is sufficient for the HAD phenotype during ASFV infection.

### CD2v-Nt N-glycosylation is essential for HAD

CD2v is a highly glycosylated protein (27, 37, 39), with an apparent molecular mass in SDS-PAGE much higher than that predicted by its amino acids content (42 kDa, see Fig. 1B, 2B and 3B). Tunicamycin is an analog of UDP-N-acetylglucosamine, which interferes with N-glycosidic linkage of core oligosaccharides to asparagine (Asn), inhibiting the first step in the biosynthesis of N-linked glycans in glycoproteins (40). The addition of tunicamycin to COS-1-infected cells inhibits CD2v-N-glycosylation, resulting in a reduction of its apparent molecular weight (37), although a specific role of CD2v-N-glycosylation in ASFV infection has not been assigned yet.

**Fig 5:**
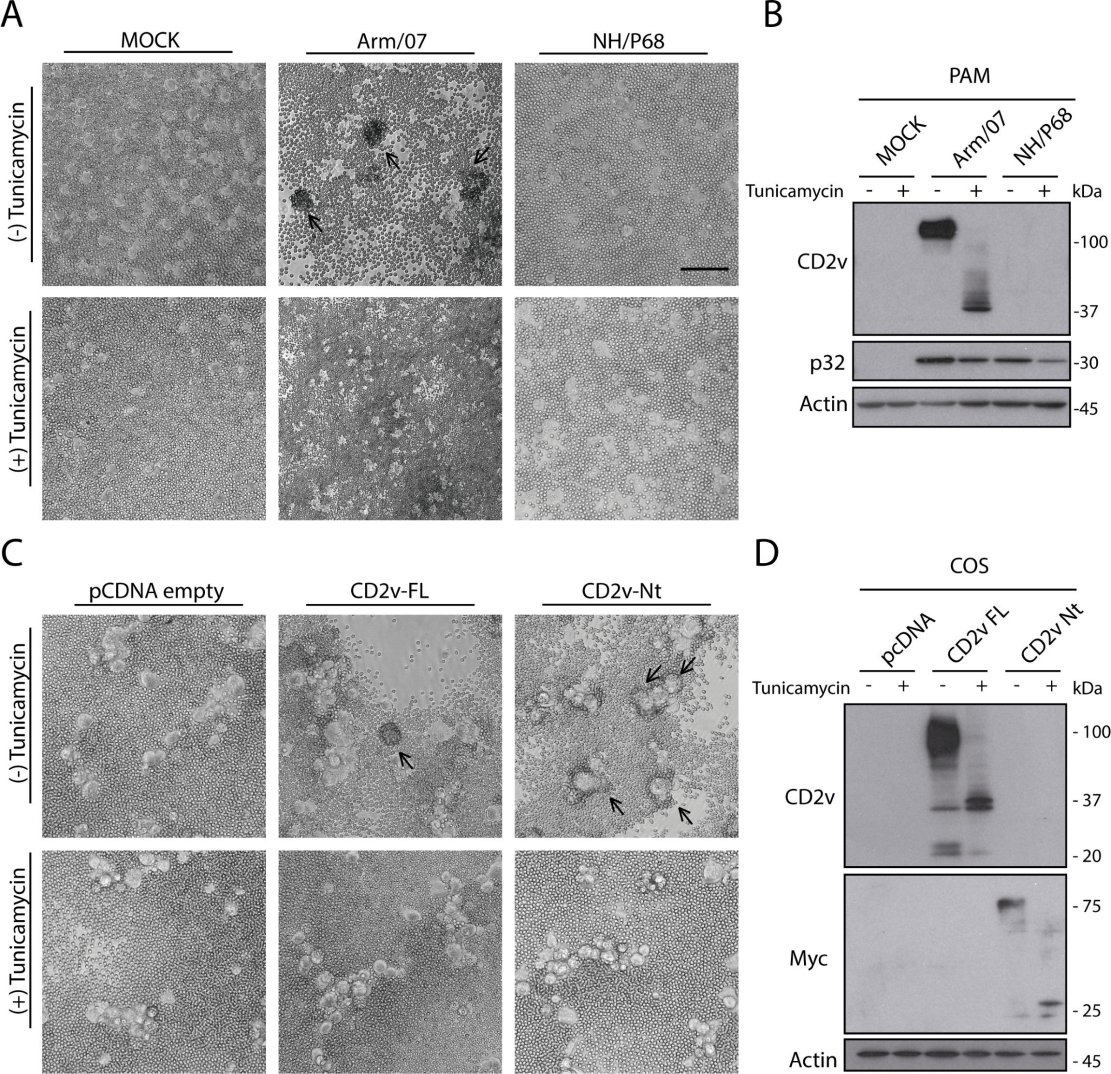
Tunicamycin treatment inhibits CD2v glycosylation and abrogates rosette formation in PAM-infected cells and in COS-1-transfected cells. (**A and B**) PAM were MOCK-infected or infected with either Arm/07/CBM/c2 (Arm/07) or NH/P68, for 16 h (MOI = 1). (**C and D**) COS-1 cells were transfected (1 µg/10^6^ cells) with empty vector (pcDNA) or expression vectors for CD2v-FL or CD2v-Nt (from Arm/07/CBM/c2 strain). At 6 hpt, cells were infected with VV-T7 (MOI = 0.5) for 16 h. Then, (**A and C**) cells were incubated with porcine erythrocytes for further 24 h before observation under light microscope; (**B and D**) cells were lysed in RIPA buffer, separated by 7 to 20% SDS-PAGE, and followed by immunoblotting with anti-ASFV CD2v, anti-ASFV p32, and anti-actin antibodies. Arrows indicate rosettes. The empty vector pcDNA control image is used in Fig. 2, 3, and 5 because they are all part of the same internally controlled experiment. A representative image (*n* = 3) is shown. Scale bar 100 µm.

With the aim to investigate a putative role of CD2v-N-glycosylation in ASFV HAD, we infected PAM with Arm/07/CBM/c2 (HAD+) or NH/P68 (HAD−) strains, treated or not with tunicamycin. At 16 hpi, porcine erythrocytes were added to the medium, and the formation of rosettes was observed 24 h later. As shown in Fig. 5A, rosette formation was prevented in Arm/07/CBM/c2-infected cells treated with tunicamycin during infection. Rosettes were neither observed in MOCK-infected cells nor in NH/P68-infected cells, independently of tunicamycin treatment. Similar results were observed in COS-1 infected with the recombinant viruses Arm-ΔCD2v-GFP, Arm-CD2v-FL*, and Arm-CD2v-Nt in the presence of tunicamycin (Fig. S2). Furthermore, tunicamycin treatment provoked that CD2v molecular weight switched from 110 kDa to 42 kDa (Fig. 5B), according to previous results (37), while no CD2v was detected either in MOCK infected or NH/P68-infected cells. Interestingly, tunicamycin treatment abrogated rosette formation in COS-1-transfected cells with either CD2v-FL or CD2v-Nt (Fig. 5C) and induced the appearance of a lower molecular weight band corresponding to non-glycosylated CD2v-FL and, more importantly, to non-glycosylated CD2v-Nt (Fig. 5D). This last result indicates that Nt domain of CD2v is highly N-glycosylated, and altogether, our data reveal the functional importance of CD2v-N-glycosylation, particularly of the Nt domain, for ASFV HAD.

### N-glycosylation of CD2v-Nt N79 and N104 residues mediates HAD

**Fig 6:**
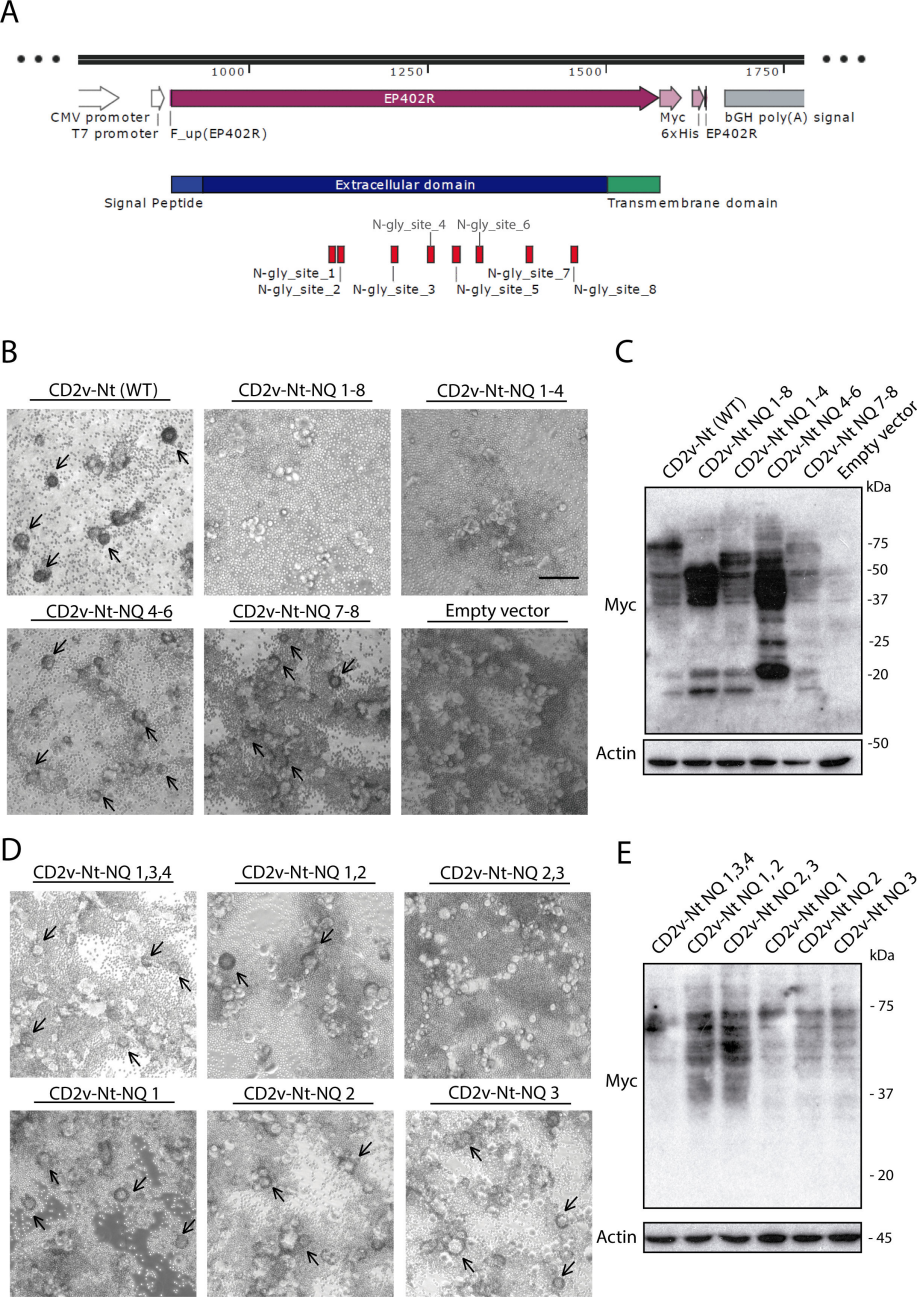
Asp (**N**) to Gln (**Q**) mutations on specific N-glycosylation sites in CD2v-Nt prevent HAD. (**A**) Diagram of the vector expressing the CD2v-Nt domain indicating the potential N-glycosylation sites to be mutated (Snapgene). COS-1 cells were transfected with empty vector or expression vectors for CD2v-Nt (WT) or CD2v-Nt-NQ mutants. Mutations were numbered as follows: N75Q:1, N79Q:2, N104Q:3, N121Q:4, N133Q:5, N144Q: 6, N167Q:7, and N188Q:8. At 6 hpt, cells were infected with VV-T7 (MOI = 0.5) for 16 h; (**B, D**) cells were incubated with porcine erythrocytes for further 24 h before observation under light microscope; and (**C, E**) cells were lysed in RIPA buffer, separated by 10% SDS-PAGE, and followed by immunoblotting with anti-myc and anti-actin antibodies. Arrows indicate rosettes. Scale bar 100 µm.

The central role of N-glycosylation for ASFV-HAD emphasizes the importance of identifying the specific residues involved in N-glycosylation, since they may mediate HAD and, in a late point, be related with virulence. *In silico* analysis identified at least eight N residues susceptible to be glycosylated in the Nt domain of CD2v from Arm/07/CBM/c2: N75, N79, N104, N121, N133, N144, N167, and N188 (displayed in Fig. 6A). In order to investigate whether the N-glycosylation of these specific residues was involved in HAD, we generated CD2v-Nt vectors in which we substitute the Asn (N) residue to Gln (Q) residue. In a first approach, we generated the following mutants: substitution of N by Q in the eight identified N residues (NQ 1–8); mutation of N75,79,104,121Q (NQ 1–4); mutation of N121,133,144Q (NQ 4–6); and mutation of N167,188Q (NQ 7–8). We then transfected these vectors together with CD2v-Nt (WT) or empty vector in COS-1 cells, followed by infection with VV-T7, addition of porcine erythrocytes, and testing rosette formation. As shown in Fig. 6B, the CD2v-Nt mutant containing the eight described N to Q mutations (NQ 1–8) completely abrogates rosette formation, indicating that some of these N residues, alone or in combination, are critical for ASFV HAD. We also observed the inhibition of rosette formation when the CD2v-Nt NQ1-4 mutant was transfected. Interestingly, mutations in N121,133,144Q (CD2v-Nt-NQ4-6 mutant) or N167,188Q (CD2v-Nt-NQ7-8 mutant) did not affect HAD. Although the expression level of CD2v-Nt-NQ mutants was similar or higher than CD2v-Nt WT, the glycosylation pattern exhibited some differences (particularly in mutants CD2v-Nt-NQ1-8 and CD2v-Nt-NQ4-6) as shown in Fig. 6C. Additional Western blot exposure from Fig. 6C can be observed in Fig. S3.

In view of these results, we generate further CD2v-Nt-NQ combinations focused on the residues N75, N79, N104, and N121, which seem to play a direct role in rosette formation: mutation of N75,104,121Q (CD2v-Nt-NQ1,3,4); mutation of N75,79Q (CD2v-Nt-NQ1,2); and mutation of N79,104Q (CD2v-Nt-NQ2,3). In addition, we generate the following individual NQ mutants: N75Q (CD2v-Nt-NQ1), N79Q (CD2v-Nt-NQ2), and N104Q (CD2v-Nt-NQ3). As shown in Fig. 6D, only the combined mutation of N79,104Q completely abrogated rosette formation, indicating that both N residues are fundamental for HAD. Other combinations containing one of these mutations, such as CD2v-Nt-NQ1,2 or CD2v-Nt-NQ1,3,4 (corresponding to N75,79Q mutation or N75,104,121Q), did not fully prevent rosette formation although it did decrease the percentage of rosettes observed (Table S3) and despite having a similar degree of glycosylation (Fig. 6E). Moreover, individual mutations N to Q in N79 or N104 showed no effect in rosette formation (Fig. 6D; Table S3).

Overall, these results indicate that N-glycosylation of both N79 (“NQ2”) and N104 (“NQ3”) in the CD2v Nt domain are essential for HAD, whereas lack of glycosylation on either one or another of these two N residues, separately, did not prevent HAD.

### Signal peptide deletion prevents glycosylation and HAD of both CD2v-FL and CD2v-Nt

**Fig 7:**
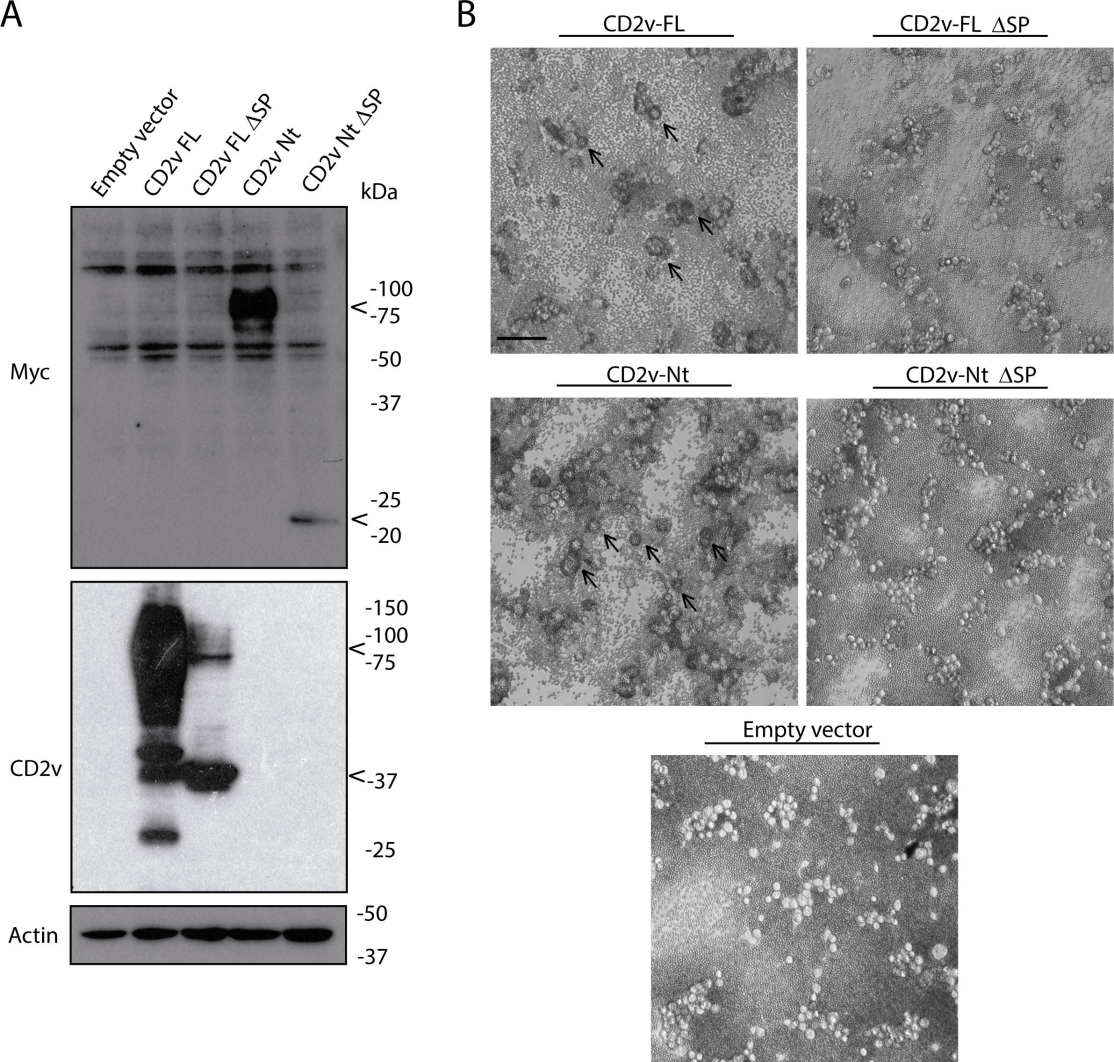
Signal peptide plays a role in Nt-CD2v glycosylation and is fully determinant for inducing HAD of both CD2v-Nt and CD2v-FL. COS-1 cells were transfected with empty vector or expression vectors for CD2v-FL, CD2v-FL ΔSP, CD2v-Nt, or CD2v-Nt ΔSP. At 6 hpt, cells were infected with VV-T7 (MOI = 0.5) for 16 h. Samples were then (**A**) lysed in RIPA buffer, separated by 10% SDS-PAGE, and followed by immunoblotting with anti-myc, anti-CD2v, and anti-actin antibodies and (**B**) incubated with porcine erythrocytes for further 24 h before observation under light microscope. Arrowheads indicate the glycosylated (100–150 kDa or 75–100 kDa for CD2v FL or Nt, respectively) and non-glycosylated (42 kDa or 20–25 kDa for CD2v FL or Nt, respectively) forms of CD2v-FL/Nt and CD2v-Nt/FL ΔSP, observed by Western blot (**A**). Arrows indicate rosettes (**B**). Scale bar 100 µm.

The *in silico* analysis of the CD2v-Nt sequence reveals several predicted putative signal peptide sequences located in the 5′ end of the Nt domain. To further understand the molecular determinants of CD2v-dependent HAD, we explore the role of these predicted signal peptide sequences of CD2v. For that, we generated a CD2v full length and CD2v-Nt lacking the signal peptide (CD2v-FL ΔSP and CD2v-Nt ΔSP) whose expression was assessed by either the anti-CD2v antibody or anti-myc antibodies, respectively. As shown in Fig. 7A (see also Fig. S3), the removal of signal peptide directly provoked a reduction of the molecular weight of CD2v-Nt, suggesting that Nt-CD2v was not glycosylated in the absence of signal peptide (see arrowheads), pointing out a role of the signal peptide in Nt-CD2v glycosylation.

Interestingly, removal of the signal peptide from CD2v-FL also results in a change in the glycosylation pattern, although a total inhibition of glycosylation was not observed, as shown in Fig. 7A. These results might attribute a role to the Ct domain (Ct-CD2v) in CD2v glycosylation. It is worth noting that the unchanged electrophoretic mobility of the Ct-CD2v expressed in the presence or absence of tunicamycin, together with the absence of potential glycosylation sites within this domain, suggests that the Ct domain is not glycosylated itself (Fig. S4), indicating that the glycosylation pattern in the CD2v-FL ΔSP corresponds exclusively to the glycosylation within the Nt-CD2v.

Similar to what Fig. 5 shows, in which lack of glycosylation leads to an impairment of HAD, no rosettes were observed in the cells transfected with CD2v-Nt ΔSP. More importantly, although glycosylation was not completely prevented when expressing CD2v-FL ΔSP, rosette formation was actually inhibited also in this case, indicating a direct role of signal peptide in HAD (Fig. 7B).

### Expression of either CD2v Nt or FL from attenuated NH/P68 strain does not lead to HAD

**Fig 8:**
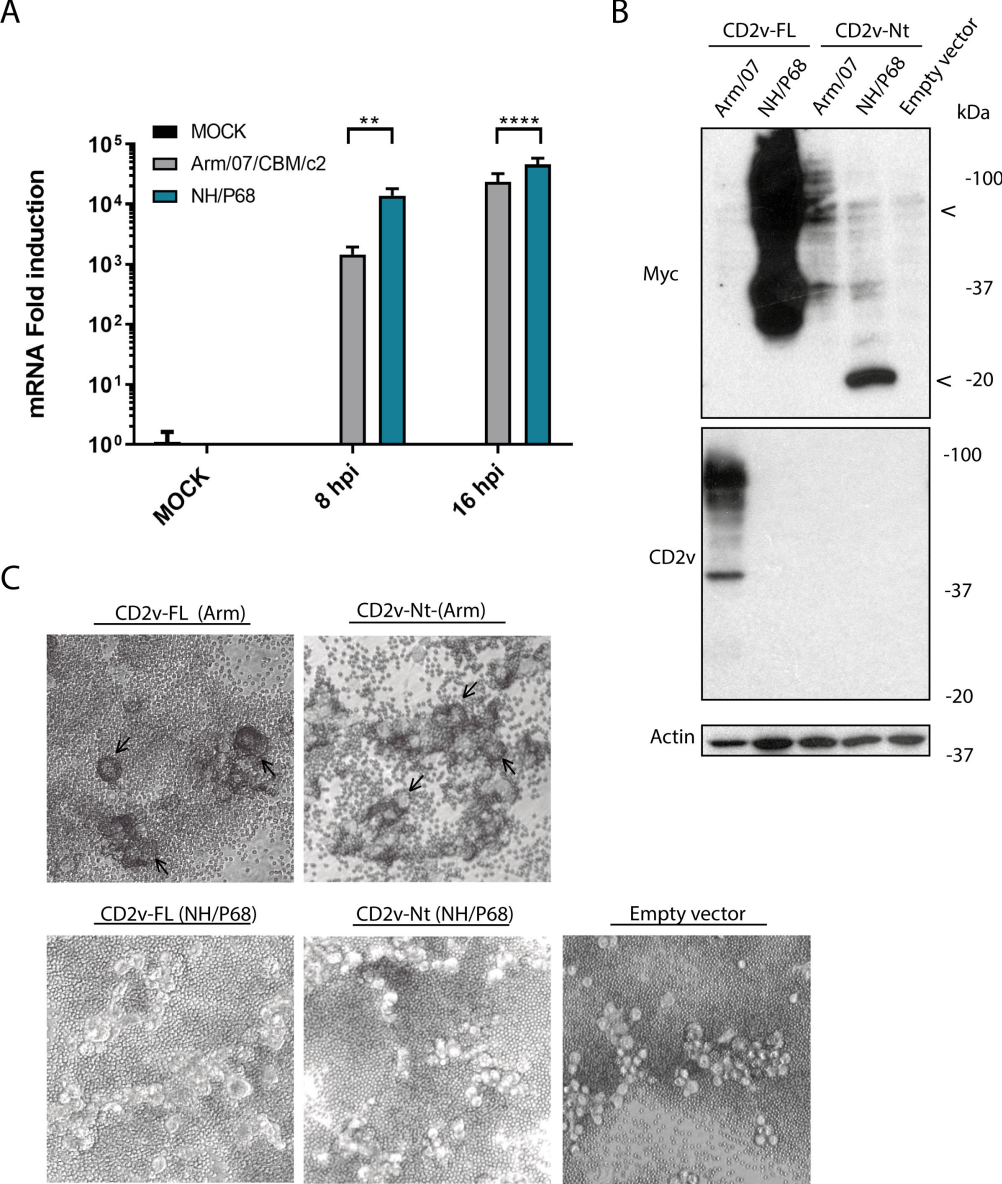
Ectopic expression of CD2v from NH/P68 is not sufficient for inducing HAD. (**A**) EP402R (coding for CD2v) mRNA was measured by RT-qPCR in PAM mock-infected or infected with either Arm/07/CBM/c2 or NH/P68 at 8 and 16 hpi, MOI = 4. (**B and C**) COS-1 cells were transfected with empty vector or expression vectors for CD2v-FL-Arm/07, CD2v-FL-NH/P68, CD2v-Nt-Arm/07, or CD2v-Nt-NH/P68. At 6 hpt, cells were infected with VV-T7 (MOI = 0.5) for 16 hpi. (**B**) They were lysed in RIPA buffer, separated by 10% SDS-PAGE, and followed by immunoblotting with anti-myc, anti-CD2v, and anti-actin antibodies; and (**C**) they were incubated with porcine erythrocytes for further 24 h before observation under light microscope. Arrowheads indicate the glycosylated and non-glycosylated forms of CD2v-Nt (Arm/07 vs. NH/P68), observed by Western blot (**B**). Arrows indicate rosettes (**C**).

Most of the known ASFV naturally attenuated viruses are non-HAD, and so, it is likely that they either do not express CD2v, or alternatively, incomplete or aberrant forms of the protein may be produced during the infection of the naturally attenuated strains. Failure to detect CD2v with our anti-CD2v antibody during NH/P68 infection does not necessarily imply that it is not being expressed, since the antibody recognizes a region of the Ct, which varies greatly between CD2v from HAD+ isolates and CD2v from NH/P68. In some cases, as in the attenuated OURT 88/3, it was suggested that EP402R (the gene coding for CD2v) had mutations that produced frameshifts, and therefore, no expression of the protein would occur (41). However, our observations reveal that the frameshift displaces the CDS from ORF+1 to ORF+3, and therefore, the resulting open reading frame corresponding to EP402R does not appear to be disrupted in neither OURT 88/3 nor NH/P68 genome. To analyze whether there is a defect at the gene expression level, we analyzed the amount of CD2v mRNA in PAMs infected with Arm/07/CBM/c2 or with NH/P68 at 8 and 16 hpi. As can be seen in Fig. 8A, CD2v (coded by EP402R gene) expression levels are even higher during infection with NH/P68 than with Arm/07/CBM/c2 infection, and so, defects at both DNA sequence and RNA expression can be ruled out.

To analyze whether CD2v from NH/P68 could be ectopically expressed and it would induce HAD, new CD2v-NH/P68 FL and Nt constructs were generated with a myc epitope included at the Ct end. The protein expression, glycosylation status, and ability to induce HAD were compared with CD2v FL and Nt constructs from Arm/07/CBM/c2, under the same conditions. Figure 8B shows, on the one hand, that unlike the CD2v Nt domain of Arm/07/CBM/c2, the CD2v Nt domain of NH/P68 is not glycosylated (see also Fig. S3 for additional Western blot exposures). Therefore, and in agreement with previous observations made regarding the importance of glycosylation for HAD, it is not surprising that the expression of the CD2v-Nt domain of NH/P68 did not result in HAD induction (Fig. 8C). On the other hand, the expression of CD2v-FL protein of NH/P68 is found to be highly glycosylated, showing a pattern that reminds to that of CD2v-FL of Arm/07/CBM/c2 (Fig. 8B). However, despite efficient expression and glycosylation, the ectopic expression of NH/P68 CD2v FL did not induce HAD (Fig. 8C).

These results indicate that even if CD2v-FL of NH/P68 was expressed during infection in its glycosylated form, it would not be able to induce rosette formation.

### Cell surface location of CD2v is not dependent on the signal peptide

In addition to glycosylation, which we have shown to be key to CD2v HAD function, intracellular localization of CD2v seems to be crucial to mediate HAD, since it is assumed that CD2v must localize in sufficient concentration on the cell surface to mediate rosette formation. The signal peptide, on the other hand, plays an important role in protein targeting and protein translocation, which could play a determining role in the surface localization of CD2v. Moreover, since CD2v of NH/P68 does not have a signal peptide and, despite being glycosylated, is not able to promote HAD, we question the possibility that its decrease or absence on the cell surface may be the cause of its defect in HAD.

**Fig 9:**
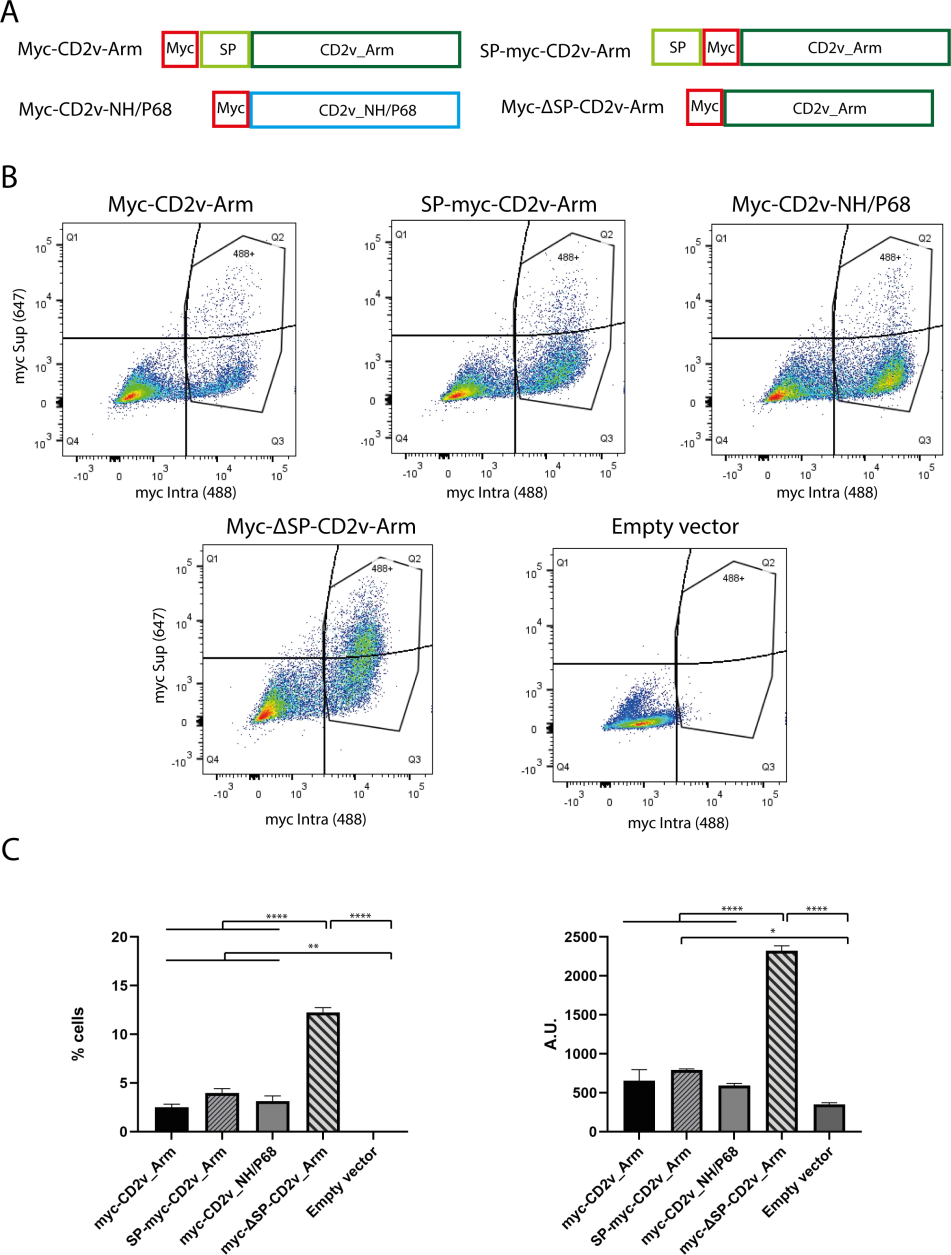
Determination of the intracellular CD2v localization by FACS. (**A**) Diagram showing the structure of CD2v vectors: myc-CD2v-Arm, SP-myc-CD2v-Arm, myc-CD2v-NH/P68, or myc-ΔSP-CD2v-Arm. (**B, C**) COS-1 cells were transfected with either myc-CD2v-Arm, SP-myc-CD2v-Arm, myc-CD2v-NH/P68, myc-ΔSP-CD2v-Arm, or empty vector (1 µg/10^6^ cells); and they were infected with VV-T7 (MOI) = 0.5 for further 16 h. Cells were then stained with the fixable Viability Dye Ghost Dye Red 780 (1 µg/mL), then with anti-myc followed by incubation with an anti-mouse Alexa Fluor-647 to localize CD2v on the cell surface (647+) and stained with anti-myc followed by incubation with an anti-mouse Alexa Fluor-488 to localize intracellular CD2v (488+). Cells were then analyzed in a FACSCanto A flow cytometer (BD Science) to determine the percentage of cells expressing CD2v in cell surface (647+) and/or expressing CD2v intracellular (488+). (**B**) Dot plot showing the distribution of CD2v+ cells in both cell surface and intracellular for each of the CD2v vector. (**C**) Percentage of cells 647+/488+ (**Q2**) (left) and MFI of surface CD2v expression (647) within cells expressing intracellular CD2v (488+) (right), for each CD2v vector. Bars indicated standard deviation (*n* = 2). Data were statistically analyzed by using one-way ANOVA (*, *P* < 0.05; **, *P* < 0.01; ****, *P* < 0.0001). B and C showed a representative experiment of three.

To determine the presence of CD2v on the cell surface, we made new constructs with the “myc” epitope at the Nt domain. Since it had been reported that the signal peptide could be hydrolyzed upon CD2v reaching the surface (39), we generated both Myc-CD2v-Arm and SP-myc-CD2v vectors. In the latter, the “myc” epitope was downstream of the signal peptide and could be detected even if the signal peptide was hydrolyzed. In addition, we generate the Myc-CD2v-NH/P68 and the Myc-ΔSP-CD2v-Arm vectors (see Fig. 9A). These constructs were used to transfect COS-1 cells that were then infected with VV-T7 as previously explained, before surface labeling with anti-myc followed by secondary antibody M-647 to detect CD2v expressed on the cell surface, then permeabilizing the cells and re-labeling with anti-myc, followed by secondary antibody M-488 to detect intracellular CD2v (see Materials and Methods).

As can be seen in Fig. 9B, two patterns of CD2v expression were observed: surface and intracellular expression (647+/488+, Q2) or only intracellular expression (647−/488+, Q3), in addition to double negative, non-transfected (647−/488−, Q4). On the one hand, we observed that the surface CD2v expression (Q2) pattern showed by the Myc-CD2v-Arm and SP-myc-CD2v-Arm constructs was very similar (Fig. 9B and C, left). This suggest that there is no, or only minor, proteolyzation of the signal peptide during CD2v localization on the cell surface. A non-significant decrease in surface CD2v detection is observed in the Myc-CD2v-Arm vs SP-myc-CD2v-Arm construct, probably due either to a proteolyzation of the signal peptide of a small part of the proteins reaching the surface or to the different sensitivity of each construct to be detected by the anti-myc.

Surprisingly, we observed no differences in surface localization between the Myc-CD2v-Arm, SP-myc-CD2v-Arm, and the Myc-CD2v-NH/P68 vectors (Fig. 9B). This would rule out the hypothesis that NH/P68-encoded CD2v is not able to reach the cell surface, even in the absence of signal peptide. Therefore, the defect of CD2v in inducing HAD does not appear to come from either a defect in glycosylation (Fig. 8B) or in its surface localization (Fig. 9B and C). Since it had been assumed that the signal peptide contained the information for protein localization, it is surprising that even in the absence of the signal peptide, CD2v from NH/P68 is able to reach the cell surface. This result is confirmed by the localization of the Myc-ΔSP-CD2v-Arm vector, whose surface detection (Q2) is significantly higher than any of the other three constructs (Fig. 9B and C, left). Furthermore, we quantified the mean fluorescence intensity (MFI) of surface CD2v (647), within cells expressing intracellular CD2v (488+) (Fig. 9C, right), confirming a significant increase in cells transfected with Myc-ΔSP-CD2v-Arm in surface CD2v intensity. This thus confirms that the signal peptide is not required for CD2v to reach the cell surface, but, curiously, its absence should favor the presence of CD2v on the cell surface. It might be hypothesized that recycling signals in the signal peptide could be involved in the CD2v traffic between the cell surface and the endosomes, explaining these unexpected results.

### CD2v-predicted signal peptides from HAD+ strains do not recover the HAD phenotype of CD2v from NH/P68

**Fig 10:**
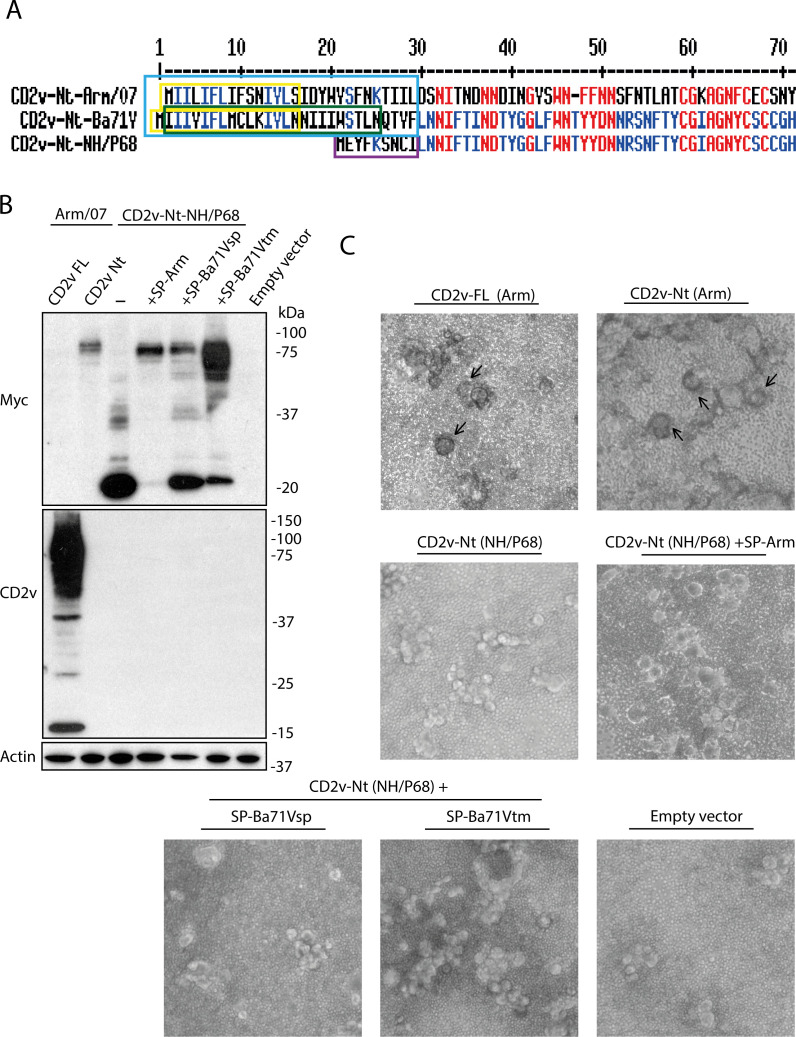
Complementation of the CD2v-Nt domain of NH/P68 with the CD2v signal peptides of either Arm/07 or Ba71V fails to recover the HAD phenotype. (**A**) Sequence comparison of CD2v Nt-terminal sequences of Arm/07/CBM/c2, Ba71V, or NH/P68 and signal peptides where appropriate. The yellow box highlights the *in silico* predicted signal peptide sequence for CD2v of Arm/07/CBM/c2 and Ba71V (SP-Arm and SP-Ba71Vsp, respectively). In the green box, the predicted transmembrane sequence in CD2v of Ba71v (SP-Ba71Vtm) is highlighted. In the blue box, the entire Nt-terminal sequence of CD2v of Arm/07 or Ba71V that does not appear in CD2v of NH/P68 is highlighted (SP-Arm_whole_ or SP-Ba71V_whole_, respectively). Finally, the purple box highlights the single sequence appearing at the Nt end of CD2v of NH/P68 that does not appear in CD2v of HAD+ strains. (**B, C**) COS-1 cells were transfected with empty vector or expression vectors for CD2v-FL-Arm/07, CD2v-Nt-Arm/07, CD2v-Nt-NH/P68, CD2v-Nt-NH/P68 + SP Arm, CD2v-Nt-NH/P68 + SP-Ba71Vsp, or CD2v-Nt-NH/P68 + SP-Ba71Vtm. At 6 hpt, cells were infected with VV-T7 (MOI = 0.5) for 16 h. Samples were then (**B**) lysed in RIPA buffer, separated by 10% SDS-PAGE, and followed by immunoblotting with anti-myc, anti-CD2v, and anti-actin antibodies and (**C**) incubated with porcine erythrocytes for further 24 h before observation under light microscope. Arrows indicate rosettes (**C**).

As mentioned above, sequences at the Nt end compatible with signal peptides have been identified in CD2v of HAD+ strains. These sequences, however, are absent in CD2v from NH/P68 and other non-HAD strains such as OURT88/3. As mentioned before, the different signal peptide sequences were determined *in silico* as indicated in the Materials and Methods section, and each of the sequences is displayed in Fig. 10A. In addition, we have performed a sequence comparison of signal peptides between different strains of genotype I (HAD+ and HAD-), genotype II, and other genotypes such as III, IV, VIII, IX, XX, or XXII, as shown in Fig. S5 (the accession numbers of these genomes are indicated in Table S4). We can observe that the signal peptide corresponding to Arm/07/CBM/c2 is highly conserved in genotype II strains and is almost identical to those from genotype IV, VIII, IX, and XX. Something similar occurs with genotype I HAD+ strains, which is highly conserved among them, including that of Ba71V, as well as in genotype III and XXII strains, with the peculiarity of genotype I strain E75, whose predicted signal peptide is shorter.

In order to investigate if the lack of the canonical signal peptide in CD2v of NH/P68 (genotype I) is involved in the lack of HAD activity, we plan new constructs by adding the CD2v signal peptide from the Arm/07/CBM/c2 strain (genotype II) or the Ba71V strain (genotype I) to the Nt of CD2v of NH/P68, which shares 100% similarity with other genotype II strains (Georgia 2007/1, SY-2 or Korea/Pig/Paju/2019) or genotype I (26544/OG10 or Benin 97/1) strains, respectively. In the case of CD2v from Ba71V (genotype I), in addition of the potential signal peptide, we identified another longer sequence as a potential transmembrane domain. For this reason, two different sequences, named SP-Ba71V-sp and SP-Ba71V-tm, were cloned. As shown in Fig. 10B (see also Fig. S3 for additional Western blot exposure), the addition of the different signal peptides produced a striking change in the CD2v-Nt glycosylation pattern of NH/P68. While CD2v-Nt of NH/P68 itself does not appear glycosylated, in contrast to CD2v-Nt of Arm/07/CBM/c2, the addition of SP-Arm, SP-Ba71Vsp, or SP-Ba71Vtm induced full or partial glycosylation of CD2v-Nt of NH/P68, respectively. However, this change in the glycosylation pattern of CD2v-Nt of NH/P68 did not lead to the recovery of the HAD phenotype (Fig. 10C).

In addition to those signal peptides tested, we investigated the effect that signal peptide in CD2v from E75 strain (genotype I) might have on the rescue of HAD phenotype of CD2v-Nt of NH/P68. In addition, we speculate that not only the signal peptide but also the unique Nt-terminal sequence of CD2v of NH/P68, compared to the analogous sequence found in genotype I HAD+ strains, could have also a role in HAD (see purple and blue boxes in Fig. S6). To assess that, we generated two new constructs: first one, complementing the CD2v-Nt of NH/P68 by adding the signal peptide of E75 (CD2vNt-NH/P68 + SP-E75); and a second one in which we exchanged the unique sequence of the Nt end of CD2v of NH/P68 for the analogous sequence found in genotype I HAD+ strains (CD2vNt-NH/P68-Seq.inter) (sequences are indicated in Fig. S6). As in the previous experiment, these constructs were transfected into COS-1 cells, together with the vectors corresponding to CD2v-Nt of Arm/07/CBM/c2, CD2v-Nt of NH/P68, and CD2v-Nt of NH/P68 + SP-Ba71Vsp or +SP-Ba71Vtm. As can be seen in Fig. S6, the addition of SP-E75 causes almost complete glycosylation of CD2v-Nt of NH/P68, very similar to the pattern observed upon the addition of SP-Ba71Vtm. However, neither CD2vNt-NH/P68 + SP-E75 nor CD2vNt-NH/P68-Seq.inter (which did not induce glycosylation) was able to recover the HAD+ phenotype (Fig. S6).

### Removal of CD2v-NH/P68 inhibitory sequence along with different CD2v signal peptides recovers hemadsorbing phenotype

**Fig 11:**
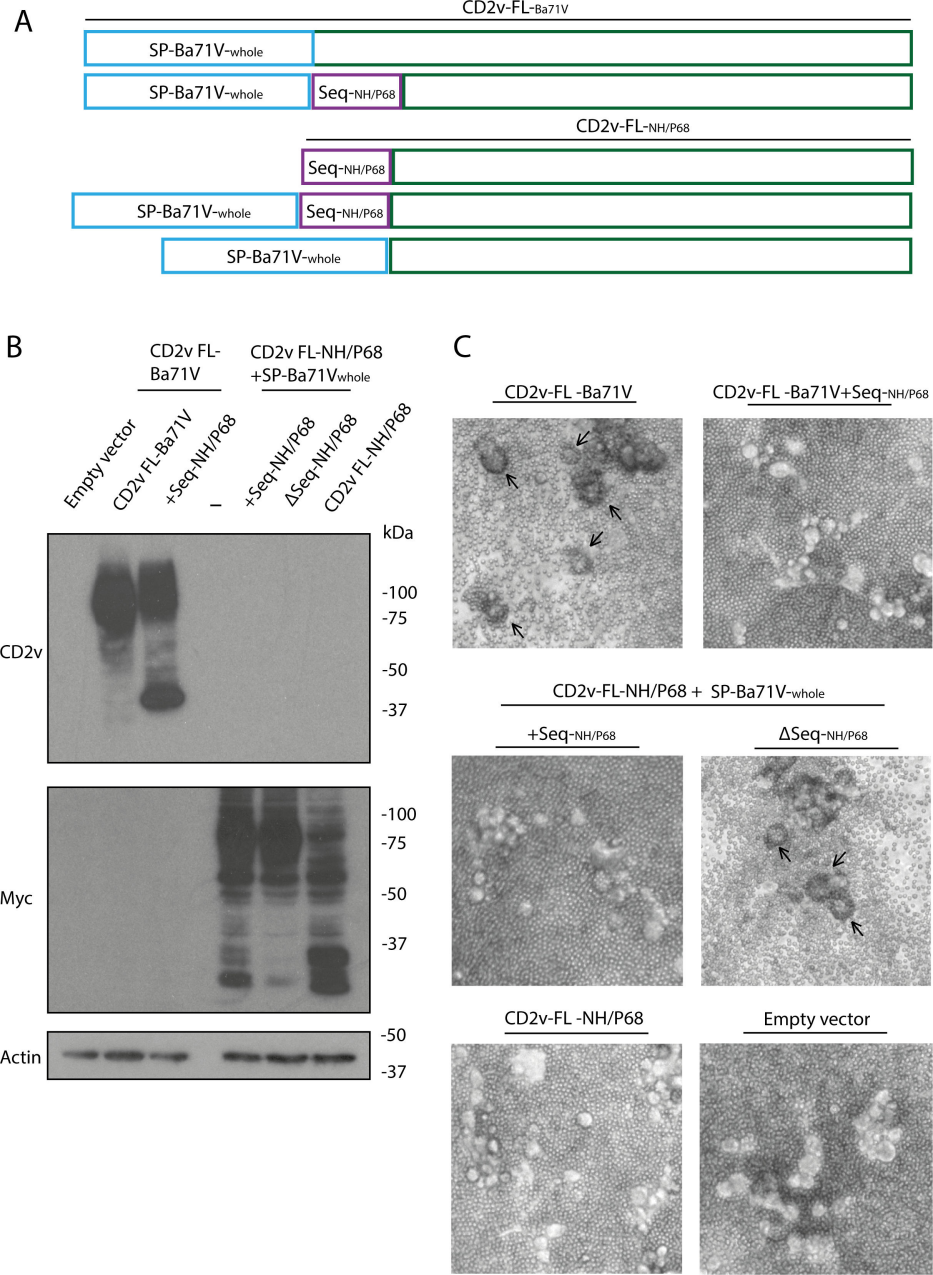
The CD2v Nt-terminal sequence of NH/P68 prevents CD2v-Ba71V-dependent HAD, and CD2v-FL-NH/P68 recovers the HAD phenotype only when this sequence is removed together with the addition of CD2v-Ba71V “whole“ signal peptide. (**A**) Diagram showing the structure of CD2v-FL-Ba71V, CD2v-FL-Ba71V + Seq-NH/P68, CD2v-FL-NH/P68 + SP-Ba71V_whole_ or CD2v-FL-NH/P68ΔSeq + SP-Ba71V_whole_. (**B, C**) COS-1 cells were transfected with empty vector or expression vectors for CD2v-FL-Ba71V, CD2v-FL-Ba71V + Seq-NH/P68, CD2v-FL-NH/P68 + SP-Ba71V_whole_, or CD2v-FL-NH/P68ΔSeq + SP-Ba71V_whole_. At 6 hpt, cells were infected with VV-T7 (MOI = 0.5) for 16 h; (**B**) they were lysed in RIPA buffer, separated by 10% SDS-PAGE, and followed by immunoblotting with anti-myc, anti-CD2v, and anti-actin antibodies and (**C**) incubated with porcine erythrocytes for further 24 h before observation under light microscope. Arrows indicate rosettes (**C**).

In order to identify the sequences able to recover the full HAD capacity of CD2v of NH/P68, new constructs including the entire sequence absent in the CD2v of NH/P68 (which included the signal peptide sequence previously identified *in silico*, from either Arm/07/CBM/c2 or Ba71V), named SP-Arm_whole_ or SP-Ba71V_whole_, respectively (Fig. 10A, blue box), were generated. Based on these vectors, we generated constructs in which the unique sequence identified at the Nt end of CD2v was removed from NH/P68 sequence (see purple box in Fig. 10A), generating the vectors CD2v-FL-NH/P68ΔSeq + SP-Ba71V_whole_ (Fig. 11A) or +SP-Arm_whole_ (Fig. S7A).

As seen in Fig. 11B and Fig. S7B, the addition of these signal peptides did not produce prominent changes in the CD2v-FL glycosylation pattern of NH/P68, which was already glycosylated. However, we observe a small increase in the glycosylated forms and a decrease in the unglycosylated forms in the absence of the “unique” sequence identified in CD2v of NH/P68 (CD2v-FL-NH/P68ΔSeq + SP- Ba71V_whole_) (Fig. 11B). Importantly, the addition of this sequence to CD2v of Ba71V (see diagram Fig. 11A) causes a pronounced decrease in the glycosylated forms of this protein, indicating that the presence of this unique sequence of CD2v of NH/P68 is involved in the CD2v glycosylation events (Fig. 11B).

Regarding HAD ability, the addition of SP-Ba71V_whole_ (Fig. 11C) or SP-Arm_whole_ (Fig S7C) to CD2v-FL-NH/P68 did not produce any rosette, indicating that the HAD phenotype could not be recovered just by adding these sequences to the whole CD2v-FL of NH/P68. However, importantly, the expression of the CD2vFL-NH/P68-Δseq + SP-Ba71V_whole_ or + SP-Arm_whole_ constructs efficiently induced rosettes, fully recovering the HAD phenotype, comparable to that obtained with CD2v-FL-Arm/07/CBM/c2 (Fig. 11C; Fig. S7C; Table S3). Not only that, but replacement of the CD2v equivalent sequence from Ba71V with the inhibitory sequence from CD2v of NH/P68 resulted in total inhibition of its ability to induce HAD (Fig. 11C). In the light of these data, we conclude that the presence of this sequence can by itself inhibit HAD.

These results indicate, on the one hand, the existence of a unique sequence in the CD2vNt of NH/P68, which, in addition to producing a decrease in the glycosylation of the protein, prevents rosette formation. And, on the other hand, that the CD2v-Nt-end sequence of HAD strains has the ability to induce HAD in CD2v-FL of NH/P68, regardless of whether it is CD2v from genotype I or genotype II strains, as long as the inhibitory sequence on CD2v of NH/P68 is not present.

Finally, we wanted to investigate what was the minimal sequence needed to recover the HAD phenotype, and whether the signal peptides predicted *in silico* (sequence indicated in Fig. 10A, yellow boxes) could complement the HAD phenotype to the NH/P68 CD2v-FL to which we removed the Nt end sequence (CD2v-FL-NH/P68-ΔSeq) demonstrated to be inhibitory for the HAD phenotype. To do this, we generated new constructs on CD2v-FL-NH/P68-ΔSeq to which we added the sequences indicated in Fig. 10A (yellow boxes), generating the vectors CD2v-FL-NH/P68-ΔSeq + SPArm, CD2v-FL-NH/P68-ΔSeq + SPBa71Vsp, and CD2v-FL-NH/P68-ΔSeq + SPBa71Vtm. As shown in Fig. S8, despite the observed glycosylation pattern, none of the three constructs were able to recover the HAD phenotype.

These results demonstrate that, to allow HAD, it is necessary not only to remove the sequence identified at the Nt end of CD2v from NH/P68 but also to add the entire sequence of the signal peptide. Importantly, our data show that the signal peptide sequences predicted *in silico*, even when they are very close to some others signal peptide sequences studied (as is the case for SP-Ba71Vtm and SP-Ba71V_whole_), are not sufficient to recover the HAD phenotype. And therefore, the integrity of the Nt terminus, both by the signal peptide sequences of CD2v from HAD+ strains and by the sequence identified as “inhibitory” in CD2v from NH/P68, is critical for HAD.

### Digestion with specific endoglycosidases suggests differences in the structure of NH/P68 CD2v glycans

In an attempt to further investigate the possible mechanisms by which CD2v from NH/P68, despite being glycosylated and localized on the cell surface, does not induce HAD, we analyzed its glycosylation pattern in comparison with that of CD2v from Arm/07/CBM/c2 by digestion with endoglycosidases H and PNGase F. Endoglycosidase H cleaves N-linked glycans between the two N-acetylglucosamine (GlcNAc) residues, but not complex glycans, while PNGase F is a glycoamidase that cleaves the bond between the Asn residue of the protein and the GlcNAc residue that joins the carbohydrate to the protein, liberating nearly all known N-linked glycans from glycoproteins (42). Digestion with these enzymes should clarify whether glycans added are incorporated at the ER or cis-Golgi, or they are more complex glycans added during traffic through the TGN. In addition, we wanted to know either whether the addition of the signal peptide to CD2v from NH/P68 or the removal of the inhibitory sequence, had effect on the digestion pattern of the glycans.

**Fig 12:**
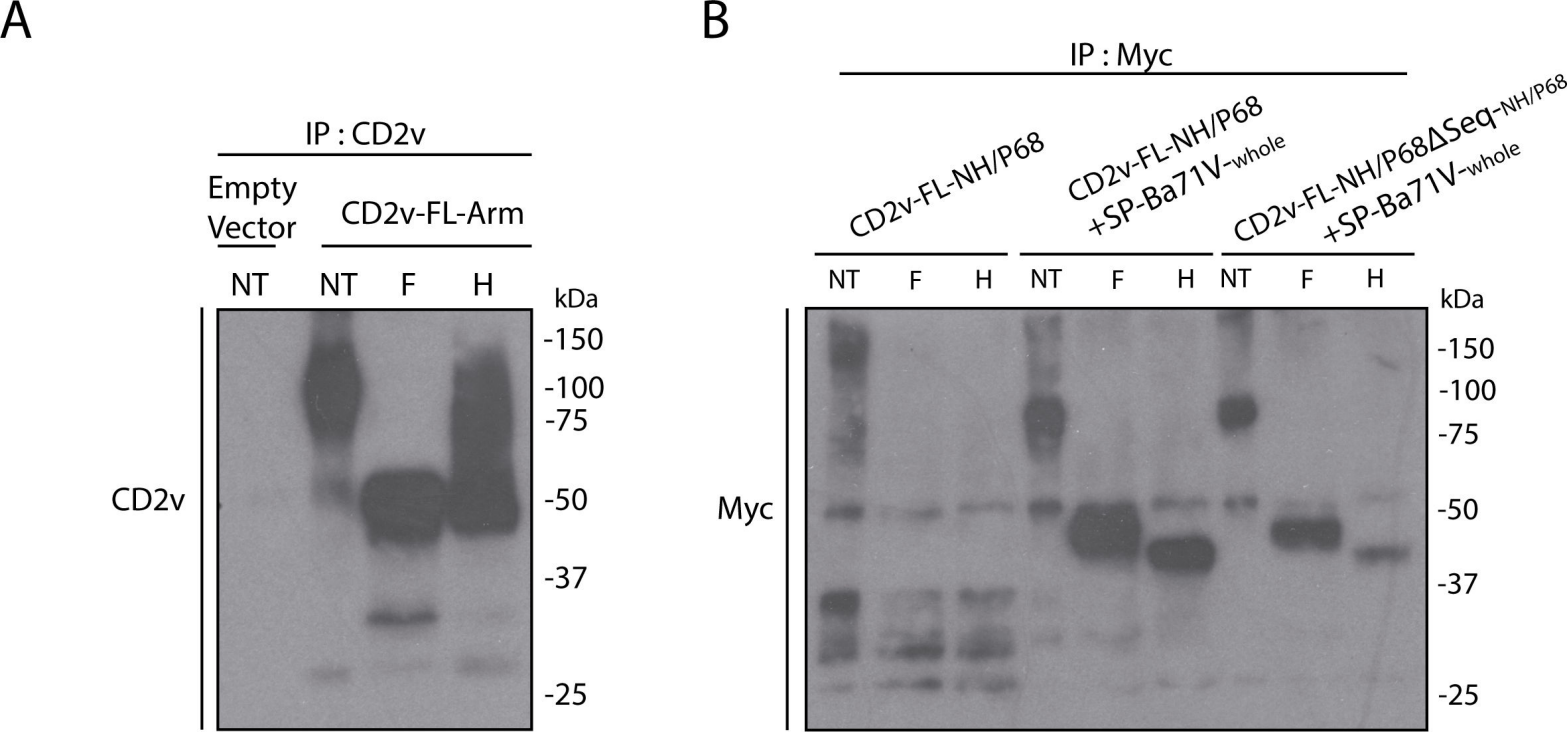
Digestion with endoglycosidases H and PNGase F shows a different pattern of CD2v glycans from NH/P68, which is modifiable by the signal peptide. COS-1 cells were transfected with empty vector or CD2v-FL-Arm (**A**), or CD2v-FL-NH/P68, CD2v-FL-NH/P68 + SP-Ba71V_-whole_ or CD2v-FL-NH/P68ΔSeq + SP-Ba71V_-whole_ (**B**). At 6 hpt, cells were infected with VV-T7 (MOI = 0.5) for 16 h and immunoprecipitated using either anti-CD2v bonded to magnetic beads Pierce Protein A/G Magnetic Beads (**A**) or Pierce Anti- anti-c-Myc Magnetic Beads (**B**). The different immunoprecipitates were eluted and non-treated (NT) or treated with either endoglycosidases PNGase-F (**F**) or Endo-H (**H**). Finally, samples were separated by 10% SDS-PAGE and followed by immunoblotting with anti-myc, anti-CD2v, and anti-actin antibodies.

For this purpose, we transfected COS-1 cells with the indicated constructs, and then, immunoprecipitation was performed with anti-CD2v or anti-myc, as appropriate. The resulting immunoprecipitated proteins were then untreated (NT) or digested with endoglycosidase H (H) or PNGase F (F), and the corresponding pattern of digestion was observed by Western blotting (Fig. 12).

As can be seen in Fig. 12A, digestion of CD2v from Arm/07/CBM/c2 with PNGase F produced a drastic reduction in its molecular weight, compatible with the digestion of most glycans, whereas digestion with endoglycosidase H produced a smaller decrease, suggesting that many of the glycosides present in CD2v from Arm/07CBM/c2 are resistant to digestion with endoglycosidase H. Interestingly, digestion with these enzymes produced quite different results on CD2v from NH/P68 (Fig. 12B), as digestion with both PNGase F and endoglycosidase H produced a virtually identical deglycosylation pattern, suggesting that there are no, or very few, glycans resistant to endoglycosidase H digestion in CD2v from NH/P68. On the other hand, CD2v of NH/P68-containing Ba71V signal peptide undergoes a different digestion pattern, showing forms resistant to digestion with both enzymes which could be related to their ability to HAD. However, the elimination of the inhibitory sequence does not seem to affect the pattern of digestion with the endoglycosidases tested (Fig. 12B).

In conclusion, it appears that CD2v of HAD+ strains versus CD2v of NH/P68 could have different nature, and this would be affecting the HAD capacity, which in turn should be determined by the presence of the signal peptide, but not by the presence or absence of the inhibitory sequence.

## DISCUSSION

The current ASFV expansion urges the development of safe and efficient vaccines, and for this, it is essential to establish the virulence factors, as well as their molecular mechanisms. Our group described for the first time the importance of the viral control of the innate immune response and particularly the regulation of several steps of the cGAS/STING (11) and the JAK/STAT (12) pathways. Numerous subsequent studies have validated this hypothesis, identifying many viral proteins involved in the control of these cellular pathways (43 – 49), some of them are directly involved in virulence (50, 51). Other genes involved in viral growth, structural genes, and unknown function have also been directly related to virulence (50, 52 – 57).

HAD is one of the features of ASFV that has been frequently related to virulence. The appearance of attenuated ASFV strains that are not hemadsorbent, both genotype I (21, 32) and genotype II (22), establishes a certain relationship between HAD and virulence. HAD is also a factor that plays a role in virulence in other pathogens, such as malaria (24). This disease is caused by the *Plasmodium falciparum* parasite, which infects erythrocytes. In hemadsorbing strains, infection causes infected erythrocytes to surround themselves with other erythrocytes, forming rosettes. The hemadsorptive phenotype is associated with severe forms of malaria and, although different models exist, no clear molecular mechanism has been described to explain *in vivo* the relationship between virulence and HAD (58 – 60). In the case of ASFV, HAD has been speculated to promote the spread of the virus (18) and might also contribute as a mechanism of immune evasion.

The identified ASFV viral factors related to HAD have been the products encoded by the EP153R (28) and EP402R (26, 27) genes. These proteins could also have other roles during viral infection such as downregulation of MHC-I, in the case of EP153R (61); or binding to cellular proteins such as AP1 (37) or be involved in the production of type I IFN, in the case of CD2v (62). Both have also been related to established serotypes based on antibody-mediated HAI, which in turn are related to cross-protection between different serotypes (15, 17). However, in this work, we have shown that CD2v itself, and in particular its Nt domain, is involved in the hemadsorbing phenotype, ruling out the role of EP153R previously proposed, and further studying the molecular determinants of CD2v in HAD. We have tested this fact not only during ectopic expression of the CD2v-Nt domain, but more important, during infection by generating a recombinant virus expressing only the CD2v-Nt domain, being the first time that functional ASFV mutants with partially modified genes have been generated. Thus, first, we have established a direct and unique relationship between HAD and CD2v, and, second, we have shown that a recombinant virus expressing only the Nt domain of CD2 is hemasorbent. We have observed differences in the percentage of rosettes between the assays with COS-1 cells (transfected or infected) compared to PAM, where the percentage is higher. While it is difficult to completely rule out the involvement of some elements unique to ASFV infection, in view of our results it seems more likely that the differences are due to cellular factors mediating HAD. Specifically, ASFV-infected COS-1 cells show a similar percentage to that of transfected COS-1 and lower than ASFV-infected PAM (Tables S2 and S3).

However, the connection between CD2v and virulence is controversial, because since there are experimental evidence pointing to this, i.e., deletion of CD2v causes total or partial attenuation of virulent strains such as Ba71 (30) or Kenya-IX-1033 (29), other reports indicated that the deletion of CD2 did not cause a change in virulence in virulent strains, as in the case of the virulent strains Georgia or Malawi (25, 31) and in the virulent Congo-v strain (63). Nonetheless, this would indicate that the genetic context of different genotypes might determine the degree of virulence vs. CD2v expression. On the contrary, CD2v has also been part of deletion combinations that have led to the attenuation of virulent strains (34, 35, 64 – 66). Thus, it seems clear that, although it may not be the only factor, CD2v and HAD may contribute to ASFV virulence.

To contribute to elucidate the molecular mechanisms involved, in this study we have demonstrated the requirement of CD2v N-glycosylation for the hemadsorbing phenotype. Because of the relationship that might exist between HAD and virulence, it is conceivable that CD2v N-glycosylation would be one ASFV virulence factor. In connection to this, N-glycosylation of HA protein, involved in influenza hemagglutination, has been related to virulence in the viral strains of IAV (67 – 69). The degree of glycosylation of this protein can have important effects on virulence and immunogenicity. N-glycosylation of viral proteins play important roles in virulence and pathogenesis in other viruses such as Dengue, Zika, HIV as well as coronaviruses (70 – 72). Furthermore, we have determined that simultaneous glycosylation of residues N79 and N104 of ASFV CD2v is a critical event for HAD, and even though the Nt domain of CD2v is highly glycosylated on other residues, the lack of glycosylation at these two residues completely prevents the hemadsorbent phenotype. While individual mutation of these two residues, preventing their glycosylation, does not result in the impairment of HAD, combinations including one of the two did have some impact on HAD (Table S3). These residues are conserved in CD2v from genotype I and genotype II, surprisingly in both HAD+ and HAD− strains. This leaves open either the direct role of these two residues in an interaction with proteins or sialic acids within erythrocytes, or their role in a particular glycosidic architecture. The fact that the modification of erythrocyte sialic acid composition affects IAV HA protein binding (73, 74) allows to speculate that these two components, CD2v N-glycosylation and erythrocyte sialic acid composition, could have also a role in ASFV virulence and pathogenesis.

Other than glycosylation, we found an additional key element in mediating CD2v-dependent HAD: the Nt-end structure, including the signal peptide sequence. The signal peptide is typically a small peptide located primarily at the Nt end of the protein that possesses information about the secretion pathway as well as the final location of the target protein [reviewed in reference (75)]. We have seen that the signal peptide sequence is quite conserved between HAD+ strains of genotype II with others of genotype IX, VIII, XX, or IV, as well as between HAD+ strains of genotype I with others of genotype III and XXII (Fig. S5). In this study, we demonstrated a direct relation between the signal peptide and N-glycosylation of CD2v. We found that the deletion of the signal peptide, both from the full-length CD2v and the Nt domain, causes a drastic decrease in glycosylation, although the mechanism by which this occurs remains to be elucidated. One scenario would be that the signal peptide could determine whether glycosylation would be co- or post-translational, which in turn could determine the architecture of N-glycosylation (76). However, CD2v from the HAD- NH/P68 strain shows a peculiar behavior regarding glycosylation, compared to CD2v from Arm/07/CBM/c2: in the attenuated strain, while the CD2v-FL is highly glycosylated, the Nt domain is not. It is intriguing that the ectopic expression of the Nt domain of CD2v of NH/P68, where potential N-glycosylation sites are located, is not glycosylated. On the contrary, the ectopic expression of CD2v-FL from Arm/07/CBM/c2 lacking signal peptide showed a very poor glycosylation pattern, which is completely abolished when Nt domain lacking the signal peptide was expressed. This would point to a role of the CD2v-Ct domain in protein glycosylation, although this domain does not glycosylate itself (Fig. S4).

In any case, and regarding HAD, it seems that glycosylation is not sufficient to accomplish the hemadsorbing phenotype, and in fact, CD2v FL expression of NH/P68, despite the high glycosylation of the protein found, is not able to induce the HAD phenotype. This fact, together with the observed expression of CD2v mRNA during infection with NH/P68, might rule out a lack of CD2v expression, but rather a defect of the protein itself for its hemadsorptive function in this strain.

To further explore the lack of HAD-inducing ability of CD2v of NH/P68, we analyzed its location at the cell surface, one of the factors believed to be key to rosette formation. The signal peptide, absent in CD2v of NH/P68, is thought to play a key role in this localization, and it was speculated that it could be proteolyzed upon reaching the plasma membrane (39). With these starting assumptions, we generated different constructs with the myc epitope to detect CD2v on the cell surface by FACS: Myc-CD2v-Arm and SP-myc-CD2v-Arm. In case that the signal peptide was proteolyzed, the “myc” epitope would also be proteolyzed in the Myc-CD2v-Arm construct, but not in the SP-myc-CD2v-Arm construct. However, surface localization data show that the detection of both constructs by the anti-myc antibody was very similar, indicating little or no proteolysis of the signal peptide.

Even more surprising is that CD2v of NH/P68 and Myc-ΔSP-CD2v-Arm mutant, both lacking signal peptide, also localize to the cell surface. The signal peptide possesses signals for protein entry into the ER and from there follow different pathways either through the Golgi or alternative pathways to reach different organelles, the plasma membrane or secretion (77). The fact that CD2v from NH/P68 is glycosylated, and also localizes to the cell surface, would imply that it is being transported to the ER and possibly to the Golgi, where it would be glycosylated, and from there to the plasma membrane, in the absence of known signal peptide. This could be explained either by the existence of further undescribed trafficking signals, by the action of some other cellular protein or due to the “viral context,” which we try to mimicry by co-infection with VV-T7.

On the contrary, while the deletion of signal peptide has an effect on CD2v glycosylation of Arm/07/CBM/c2, it does not inhibit its localization on the cell surface, but rather produces a positive effect on the number of CD2v molecules on the surface. This could imply the existence of recycling between the plasma membrane and endomembranes, which would also explain the relatively low percentage of cells expressing surface CD2v detected in CD2v from Arm/07/CBM/c2. Since deletion of the signal peptide increases the permanence of CD2v on the cell surface, this would imply that recycling signals would exist within the signal peptide. However, canonically, recycling signals are usually located in the intracellular part of the protein, and in fact, we previously described that CD2v interacts with AP-1, a cell trafficking regulator, through its intracellular domain (37). More research is needed to unravel the mechanisms and molecular determinants of CD2v trafficking through the cell and how this relates to HAD and possibly to ASFV virulence.

Initially, the lack of signal peptide in CD2v from NH/P68 seemed to be responsible for the HAD- phenotype, and therefore, complementation studies were performed by generating different constructs of CD2v-Nt and FL-NH/P68 in which different *in silico* predicted CD2v signal peptides from HAD+ strains of different genotype (I or II) were added. However, none of the predicted signal peptides produced any recovery of the HAD phenotype.

It is noteworthy that we have found a sequence next to the predicted signal peptide, which is different among HAD vs. non-HAD strains. Significantly, we found that HAD only occurs when this particular sequence was deleted from non-HAD NH/P68. Importantly, the identification of this sequence in NH/P68-CD2v, as a functional inhibitory for HAD, was totally unexpected. The existence of this sequence had not been ever reported and not either identified any particular *in silico* motif on it. Furthermore, we have found that if we add this inhibitory sequence to CD2v from HAD+ strains, the HAD+ phenotype reverted to a HAD- phenotype. The mechanism by which this occurs is unknown, although we found a relationship between increased glycosylation and the absence of this sequence and conversely, so it could be speculated that this sequence inhibits glycosylation of certain residues that are particularly involved in HAD. It might be also involved in the correct folding of the protein, which may affect the rate of progression through the secretory pathways.

We next investigated if we could restore HAD ability by deleting the inhibitory sequence from NH/P68 CD2v and substituting it by the predicted signal peptide from HAD+ strains. However, we also found that the analogous sequence next to signal peptide found in HAD+ strains was also necessary for HAD. Thus, all these elements are needed to render the NH/P68 CD2v hemadsorbent: (i) the predicted signal peptide found in HAD+ strains, (ii) the CD2v “analog” sequence from HAD+ strains found next to signal peptide, and (iii) the lack of the inhibitory sequence of non-HAD strain. It remains to be determined whether the “analogous sequence” in HAD+ strains is part of the signal peptide and whether it plays another role in the architecture of the protein or play a specific role in HAD.

Furthermore, we have seen that, in these conditions, CD2v complementation of NH/P68 (genotype I) occurs similarly with CD2v-signal peptides from either genotype I or genotype II strains. CD2v together with EP153R mediate serotypes (15), and it has been shown that these may be important in mediating homologous protection, without affecting the HAD ability (16). Our data indicate that signal peptides of different genotypes may complement the hemadsorbing capacity of CD2v. It remains to be studied whether this could somehow affect the ability of vaccine prototypes to protect against homologous and/or heterologous isolates.

Finally, the nature of the glycans present in CD2v from NH/P68 vs CD2v from HAD+ strains has been studied by digestion with specific endoglycosidases. These preliminary results reveal differences in the digestion pattern between CD2v from NH/P68 and CD2v from Arm/07/CBM/c2, indicating that only in the latter, glycans resistant to endoglycosidase H and PNGase F exist. Interestingly, whereas the addition of the signal peptide from CD2v of Ba71V to CD2v from NH/P68 modifies the digestion pattern, the presence or absence of the inhibitory sequence does not have effect on this. It seems clear that the presence and nature of CD2v glycans will determine the interaction with the erythrocyte and the formation of rosettes and therefore the HAD phenotype and its pathological implications. This opens new insights that we plan to explore in the near future.

HAD is a phenomenon likely impacted by cell-type-specific functions, such as correct folding, appropriately glycosylation and transport to the cell surface for interaction with receptors present on erythrocytes.

In conclusion, this study lays the molecular basis for ASFV HAD and identifies multiple key players in this phenomenon. It remains to be determined the direct relationship of each of these elements in ASFV virulence and pathogenesis, and how this knowledge could be applied to vaccine development, with molecular modifications that may increase the immunogenicity and/or decrease the virulence of new or existing LAV prototypes, or even their application in future subunit vaccines.

## MATERIALS AND METHODS

### Cells and viruses

Pulmonary alveolar macrophages (PAM) were obtained by bronchoalveolar lavage as previously described (14) and were cultured in Dulbecco’s modified Eagle medium (DMEM) supplemented with 2 mM L-glutamine, non-essential amino acids, 100 U/mL gentamicin, and 10% porcine serum. COS-1 cells from African green monkey kidney were obtained from the American Type Culture Collection (ATCC) and grown in DMEM supplemented with 2 mM L-glutamine, non-essential amino acids, 100 U/mL gentamicin, and 5% fetal bovine serum (FBS) (Invitrogen Life Technologies). The ASFV strain NH/P68 (21), E70, Arm/07/CBM/c2, and Arm/07/CBM/c4 (36) were propagated in PAM and titrated by TCID50 in COS-1 cells and labeling with anti-p32 antibody. VACV-T7 (VV-T7) virus was generated elsewhere (78) and kindly provided by Dr. Alcami.

### Cloning of expression vectors

Expression vectors generated were derived from pcDNA3.1(+) or pcDNA3.1(+)/myc-hisA (Thermo Fisher Scientific), except for pcDNA-HA-EP153R that was derived from pcDNA-HA, which was made by introducing HA epitope to pcDNA-3 (Invitrogen) previously digested by BamHI and NotI (37). pcDNA-CD2v-Ba71V (37) and pcDNA-HA-EP153R were generated by PCR amplification to insert the full-length EP402R gene (coding for CD2v) or EP153R into pcDNA or pcDNA-HA, respectively. For EP153R, we add a BamHI site to the 5′ end and a NotI site to the 3′ end. PCR was performed by Phusion High-Fidelity PCR Master Mix (Thermo Fisher Scientific, MA, USA) from a lysate of cells infected with ASFV Ba71V using the following primers 5′-AGCGCCGATCCTATTTTAAGAAAAAATACA-3′ and 5′-TGCGCGCGGCCGCTTATTTACCACAAATAA-3′ and then ligated with Quick Ligase (New England Biolabs, MA, USA) into the corresponding previous digested vector.

The next following vectors have been generated by InFusion technology (Clontech) for what we designed specific oligos to amplify the viral genes or different combinations and to linearize the vector pcDNA3.1(+) or pcDNA3.1(+)/myc-hisA by PCR using the Phusion High-Fidelity PCR Master Mix (Thermo Fisher Scientific, MA, USA). Original viral DNA was obtained from the Arm/07/CBM/c2 ASFV virulent strain (36) or the NH/P68 ASFV-attenuated strain (21). The amplifications obtained were purified by means of a purification kit (Biotools) and ligated by InFusion (Clontech). The products were used to transform competent *Escherichia coli* DH5α, and from the obtained colonies, its DNA plasmid was extracted (Speedtools Plasmid DNA purification kit, Biotools) and sequenced (Macrogen).

The vectors and their specific oligo probes used for cloning are the following (Table 1).

**TABLE 1:** Oligos and templates used for cloning of the indicated vectors

Vector	Primers	Templates
pcDNA-CD2v_Arm	5′-GCTAGAGCTCGCTGATCAGC-3′ 5′-TGGGTCTCCCTATAGTGAGTCG-3′ 5′-CTATAGGGAGACCCAATGATAATACTTATTTTTTTAATATTTTCTAACATAGTTTTAAGTATT-3′ 5′-TCAGCGAGCTCTAGCTTAAATAATTCTATCTACGTGAATAA GCGAAATATTTTGGG-3′	pcDNA-CD2v-Ba71V DNA from ASFV Arm/07/CBM/c2
pcDNA-CD2v-Nt-myc [CD2v-Nt (Arm)]	5′-TAAGCTAGAGCTCGCTGATCAG-3′ 5′-TAAAAAAGTTATGATGGATATAAGAAGAATACTTATTGTTATCCC-3′ 5′-ATCATAACTTTTTTAGAACAAAAACTCATCTCAGAAGAGGATCT-3′ 5′-GCGAGCTCTAGCTTAATGGTGATGGTGATGATGACC-3′	pCDNA-CD2v_Arm pcDNA-A238L-myc-his
pcDNA-CD2v-Ct [CD2v-Ct (Arm)]	5′-GCTAGAGCTCGCTGATCAGC-3′ 5′-TGGGTCTCCCTATAGTGAGTCG-3′ 5′-CTATAGGGAGACCCAATGTTATATTATATTCCATTAAGCATCATAATTGGGA-3′ 5′-TCAGCGAGCTCTAGCTTAAATAATTCTATCTACGTGAATAAGCGAAATATTTTGGG-3′	pCDNA-CD2v_Ba71v DNA from ASFV Arm/07/CBM/c2
pFL-ΔCD2v-p72GFP	5′-TATGTACTATATATTAATTATTTAACCTTT CAAGC-3′ 5′-TTTATGAACATATGTTTTATAATATAGTATCAAAAAC-3′ 5′-ACATATGTT CATAAAGACATTGATTATTGACTAGT TATTAATAG-3′ 5′-AATATATAGTACATACCA TAGAGCCCACCG-3′	pFL_CD2v pEGFP_E3
pFL-CD2v-Nt	5′-CTTCTGAGGCGGAAAGAACCA-3′ 5′-AACGCGTATATCTGGCCCG-3′ 5′-CCAGATATACGCGTTGTTTGAAAAAAAAA ATAGATGATTATAGTATATTAATAATTGG-3′ 5′-TTTCCGCCTCAGAAGTTTTGG GAACTGTGGGCCTC-3′	pcDNA3.1 (+) ASFV_Georgia2007
pFL-CD2v-FL	5′-CTTCTGAGGCGGAAAGAACCA-3′ 5′-AACGCGTATATCTGGCCCG-3′ 5′-CCAGATATACGCGTTGTTTGAAAAAAAAAATAGATGATTATAGTATATTAATAATTGG-3′ 5′-TTTCCGCCTCAGAAGTTTTGGGAACTGTGGGCCTC-3′	pcDNA3.1 (+) DNA from ASFV Arm/07/CBM/c2
pcDNA-CD2v-NtΔSP-myc (CD2v-NtΔSP)	5′-GAACAAAAACTCATCTCAGAAGAGGATCT-3′ 5′-CATTGGGTCTCCCTATAGTGAGTC-3′ 5′-TAGGGAGACCCAATGATTGATTATTGGGTTAGTTTTAATAAAACAATAATTTTAGATAGTAATAT-3′ 5′-GATGAGTTTTTGTTCTAAAAAAGTTATGATGGATATAAGAA-3′	pcDNA-CD2vNtTM-myc pcDNA-CD2vNtTM-myc
pcDNA-CD2v-FL-NH/P68-myc (CD2v-FL -NH/P68)	5′-GAACAAAAACTCATCTCAGAAGAGGATCT-3′ 5′-TGGGTCTCCCTATAGTGAGTCG-3′ 5′-CTATAGGGAGACCCAATGGAGTACTTTAAATCAAACTGTATTTTAAATAATATTTTTACAA-3′ 5′-GATGAGTTTTTGTTCGGTGGAGGACAGGTTTGGG-3′	pcDNA-CD2vNtTM-myc ASFV_NH/P68
pcDNA-CD2v-Nt-NH/P68-myc (CD2v-Nt- NH/P68)	5′-GAACAAAAACTCATCTCAGAAGAGGATCT-3′ 5′-TGGGTCTCCCTATAGTGAGTCG-3′ 5′-CTATAGGGAGACCCAATGGAGTACTTTAAATCAAACTGTATTTTAAATAATATTTTTACAA-3′ 5′-GATGAGTTTTTGTTCTAATACAGATATGATTGAAATAAAAATACCTATTATTAATCCACTCACA-3′	pcDNA-CD2vNtTM-myc ASFV_NH/P68
pcDNA-CD2v-Nt-NH/P68 + SPAmrmyc (CD2v-Nt-NH/P68 + SPArm)	5′-GAGTACTTTAAATCAAACTGTATTTTAAATAATATTTTTACAATTAATGA-3′ 5′-CATGGTAATAGCGATGACTAATACGTAGA-3′ 5′-ATCGCTATTACCATGGTGATGCGGTTT-3′ 5′-TGATTTAAAGTACTCACTTAAAACTATGTTAGAAAATATTAAAAAAATAAGTATTATCATTGGG-3′	pcDNA-EP402R-Nt-NHV-myc pCDNA-CD2v_Arm
pcDNA-CD2v-Nt-NH/P68 + SPBa71Vsp-myc (CD2v-Nt-NH/P68 + SP-Ba71Vsp)	5′-GAGTACTTTAAATCAAACTGTATTTTAAATAATATTTTTACAATTAATGA-3′ 5′-CATGGTAATAGCGATGACTAATACGTAGA-3′ 5′-ATCGCTATTACCATGGTGATGCGGTTT-3′ 5′-TGATTTAAAGTACTCGTTTAAAACTATTTTTAAACACATTAAAAAAATAACTATTATTATCATGG-3′	pcDNA-EP402R-Nt-NHV-myc pCDNA-CD2v_Ba71v
pcDNA-CD2v-Nt-NH/P68 + SPBa71Vtm-myc (CD2v-Nt-NH/P68 + SP-Ba71Vtm)	5′-GAGTACTTTAAATCAAACTGTATTTTAAATAATATTTTTACAATTAATGA-3′ 5′-CATGGTAATAGCGATGACTAATACGTAG-3′ 5′-ATCGCTATTACCATGGTGATGCGGTTT-3′ 5′-TGATTTAAAGTACTCATTTAAAGTACTCCATATTATAATATTGTTTAAAACTATTTTTAAACACA-3′	pcDNA-EP402R-Nt-NHV-myc pCDNA-CD2v_Ba71v
pcDNA-CD2v-Nt-NH/P68 + SPE75myc (CD2v-Nt-NH/P68 + SP-E75)	5′-ATGTGTTTAAAAATAGTTTTAAACAATATTATAATATGGAGTACT-3′ 5′-CATGGTAATAGCGATGACTAATACGTAGA-3′ 5′-ATCGCTATTACCATGGTGATGCGGTTT-3′ 5′-TATTTTTAAACACATTGGGTCTCCCTATAGTGAGT-3′	pcDNA-CD2vNt-NHV + SP_Ba71V_tm pcDNA-EP402R-Nt-NHV-myc
pcDNA-CD2v-Nt-NH/P68-Seq.inter	5′-TTTTATATCATAATTTTTATTGTGAGTGGATTAATAATAGGTAT-3′ 5′-CATTGGGTCTCCCTATAGTGAGT-3′ 5′-TAGGGAGACCCAATGTGGAGTACTTTAAATCAAACTGTATTTTTAAATAATATTTTTACAA-3′ 5′-AATTATGATATAAAATAATGTGGATAAATAATTTTGATATTTTAATATTGGATTAGTAC-3′	pcDNA-EP402R-Nt-NHV-myc pCDNA-CD2v_Ba71v
pcDNA-myc-CD2v-NH/P68	5′- TAATGAGTTTAAACCCGCTGATCAG-3′ 5′- CAGATCCTCTTCTGAGATGAGTTTTTG-3′ 5′- TCAGAAGAGGATCTGGAGTACTTTAAATCAAACTGTATTTTAAATAATATTTTTACAATTAATG-3′ 5′- GGTTTAAACTCATTAGGTGGAGGACAGGTTTGGG-3′	pcDNA-myc-CD2v-Arm pcDNA-CD2v-FL-NH/P68-myc
pcDNA-myc-ΔSP-CD2v-Arm	5′- TAGGGAGACCCAATGGAACAAAAACTCATCTCAGAAGAGGAT-3′ 5′- GATGAGTTTTTGTTCCATTGGGTCTCCCTATAGTGAGTC-3′	pcDNA-SP-myc-CD2v-Arm
pcDNA-CD2v-FL-Ba71V + Seq-_NH/P68_	5′- TTTTATATCATAATTTTTATTGTGAGTGGATTAATAATAGGTAT-3′ 5′- TATTATAATATTGTTTAAAACTATTTTTAAACACATTAAAAAAATAAC-3′ 5′- AACAATATTATAATAATGGAGTACTTTAAATCAAACTGTATTTTAAATAATATTTTTACAAT-3′ 5′- AATTATGATATAAAATAATGTGGATAAATAATTTTGATATTTTAATATTGGATTAGTAC-3′	pcDNA-CD2v-Ba71V pcDNA-CD2v-FL-NH/P68-myc

pcDNA-CD2v-Nt-NQ-1-myc and pcDNA-CD2v-Nt-NQ-2-myc were generated from pcDNA-CD2v-Nt- myc by directed mutagenesis by InFusion technology (Clontech) using the following primers, respectively:

5′-CAATATATCAAATAACAAATAATTGTAGCTTAACTATTTTTCCTCATA-3′, 5′-GTTAAGCTACAATTATTTGTTATTTGATATATTGATGTACTATAATTAG-3′; and 5′-CAATATATAATATAACAAATCAATGTAGCTTAACTATTTTTCC-3′, 5′-GTTAAGCTATGATTTGTTATATTATATATTGATGTACTATAATTAG-3′.

pcDNA-CD2v-Nt-NQ1-8-myc, pcDNA-CD2v-Nt-NQ1-4-myc, pcDNA-CD2v-Nt-NQ4-6-myc, pcDNA-CD2v-Nt-NQ7-8-myc, pcDNA-CD2v-Nt-NQ1,3,4-myc, pcDNA-CD2v-Nt-NQ1,2-myc, pcDNA-CD2v-Nt-NQ2-3-myc, pcDNA-CD2v-Nt-NQ-3-myc and pcDNA-CD2v-FLΔSP, pcDNA-myc-CD2v-Arm and pcDNA-SP-myc-Arm were generated *in vitro* and purchased by Geneart (Invitrogen, MA, USA).

### Transfection, infection, and rosette observation

Specified expression vectors were used for transfection using Metafectene (Biontex, Germany) manufacture indications, using the indicated plasmid concentration. Briefly, DNA and metafectene were incubated in Optimem (Gibco, Thermo Fisher Scientific, MA, USA) at 1:3 proportion for 15 min at RT. Then, the mixture was added to COS-1 cells previously washed in Optimem and incubated for further 4–6 h. After that, cells were infected with VV-T7. For that, viral adsorption to cells was performed during 60 min at multiplicity of infection (MOI) = 0.5, followed by wash and further incubation with DMEM and 2% fetal bovine serum (FBS) for 16 h. When indicated, tunicamycin (Boehringer Mannheim, Germany) was added during adsorption and infection (5 µg/mL).

When specified, fresh porcine erythrocytes were added to transfected/infected COS-1 cells in DMEM 2% FBS (2 µL/mL). Porcine erythrocytes were obtained from heparinized porcine blood, which was incubated 1/5 with 6% dextran in phosphate-buffered saline (PBS) for 1 h. After incubation, the red (lower) phase was taken and diluted 1/2 in PBS; 24 h after the addition of erythrocytes to transfected-infected COS-1 cells, the eventual formation of rosettes was observed under Leica DM IL LED Inverted Laboratory Microscope (Leica, Germany), and images were taken using Leica Application Suite (LAS) v4.13. The number of cells with rosettes with respect to the total number of cells was quantified using ImageJ, by means of the “Couter cells” Plugin.

### Design and generation of CRISPR-Cas9 vectors for the generation of the recombinant viruses Arm-ΔCD2v-GFP, Arm-CD2v-Nt, and Arm-CD2v-FL*

Two types of vectors were used: (i) one derived from pSpCas9(BB)-2A-Puro (PX459), in which the nuclear localization sequence (NLS) has been deleted (pSpCas9(BB)ΔNLS-2A-Puro); and (ii) a pcDNA3.1-derived vector containing the flanking sequences of the target gene (EP402R), surrounding the marker gene EGFP derived from the pEGFP-E3 vector (pFL-ΔCD2v-GFP), or pcDNA3.1-derived vector containing the flanking sequences of the target gene (EP402R), surrounding the Nt-domain of CD2v (pFL-CD2v-Nt) of the full-length CD2v (pFL-CD2v-FL).

For the pSpCas9(BB)ΔNLS-2A-Puro vectors, specific gRNAs were cloned to interrupt either the EP402R gene or the EGFP gene. For the design of the gRNA, we used Protospacer. The designed gRNA sequences were as follows: 5′-TCTTCATTAGATTCAGGTGG-3′ and 5′-GCTAGCTACATGTGGAAAAGC-3′ for EP402R gene, and 5′- GTAGGTGTCATTCTATTCTG-3′ and 5′-GATTAATAGTAATCAATTACG-3′ for EGFP cassette, which were cloned into the pSpCas9(BB)ΔNLS-2A-Puro vector following the procedure described in Ran et al. (79), generating the following vectors: pSpCas9(BB)ΔNLS-2A-Puro_EP402R-gRNA-0 and pSpCas9(BB)ΔNLS-2A-Puro_EP402R-gRNA-8 and pSpCas9(BB)ΔNLS-2A-Puro_CMV-gRNA and pSpCas9(BB)ΔNLS-2A-Puro_bGH-gRNA.

For the generation of pcDNA-derived vectors, we used pcDNA3.1 vector as backbone using InFusion (Clontech), using the oligos and procedures explained in the above section.

### Generation of recombinant virus Armc4-ΔCD2v-GFP, Armc4-CD2v-Nt, and Armc4-CD2v-FL* by CRISPR-Cas9 technology

The recombinant viruses were generated as previously described (80). We first generated the Armc4-ΔCD2v-GFP virus, and after purification and further genetic characterization, we used Armc4-ΔCD2v-GFP virus as a backbone for the generation of Armc4-CD2v-Nt or Armc4-CD2v-FL*.

Briefly, COS-1 cells were co-transfected with specific pSpCas9(BB)ΔNLS-2A-Puro gRNA (2 µg) together with the respective pcDNA-derived vector (2 µg) with FuGene HD (Promega); 24 h post-transfection, puromycin (Sigma) was added to the media of transfected cells (10 µg/mL). After 48 h, transfected cells were infected at two different MOI (1 and 0.1) with Arm/07/CBM/c4 ASFV strain to generate Armc4-ΔCD2v-GFP, or with Armc4-ΔCD2v-GFP to generate Armc4-CD2v-Nt or Armc4-CD2v-FL*. At 5 days post-infection (dpi), the cells and the medium were collected and conserved at −80°C.

### Isolation of recombinant viruses from parental viruses by plaque isolation

The recombinant viruses were isolated as previously described (80). Collected recombinant virus was used to infect COS-1 cells. After 1 h 30 min of viral adsorption, the inoculum was removed, and DMEM 2% fetal bovine serum with carboxymethylcellulose (CMC) was added. Four to seven days post infection (dpi) viral plaques appeared and were identified by fluorescent microscopy (GFP+ for Armc4-ΔCD2v-GFP, GFP- for Armc4-CD2v-Nt and Armc4-CD2v-FL*). Recombinant plaques were collected by sterile tips conserved at −80°C. After three freeze/thaw cycles, extracted virus are used to infect new COS-1 cells by using the same procedure. During the isolation procedure, parental contaminant detection was checked by PCR. For that, 10 µL of the isolated plaque was digested with proteinase K (Sigma) in 1.5 mM MgCl2, 50 mM KCl, 0.45% Tween20, 0.45% NP40 and 10 mM TrisHCl pH8 buffer, incubated 30 min at 45 °C and 15 min at 95 °C. The digested isolated plaque was used as a DNA template for PCR to detect the presence of recombinants and parental viruses. The oligos used are listed in Table 2.

**TABLE 2:** Oligos used for the detection of recombinant and parental viruses by PCR

Oligos to detect	
GFP (Armc4ΔCD2v-GFP virus)	5′-ACATGGTCCTGCTGGAGTTC-3′ 5′-GCCTGAAATACCAGAAAGAGAAGAC-3′
CD2v (Armc4 wild-type and recombinant viruses Armc4-CD2v-Nt and Armc4-CD2v-FL*)	5′-CCATTAAGCATCATAATTGGGATAAC-3′ 5′-GCCTGAAATACCAGAAAGAGAAGAC-3′

When no parental virus was detected by PCR, virus was grown for DNA extraction and next-generation sequencing (NGS) analysis.

### Viral DNA extraction for NGS analysis

Viral DNA extraction of recombinant virus for NGS analysis was performed as described previously (36, 80). Briefly, recombinant viruses were grown in six to eight P100 pre-confluent COS-1 cells. After 3–4 dpi, the supernatant containing extracellular virions was collected and centrifuged at 7,000 rpm o/n at 4°C. The pellets were resuspended in cold, filtrated 10 mM Tris-HCl, pH 8.8, and treated with 0.25 U/µL DNAse I (Sigma), 0.25 U/µL nuclease S7 (Sigma), and 20 µg/mL RNAse A (Promega) in 800 mM Tris-HCl, pH 7.5, 200 mM NaCl, 20 mM CaCl2, and 120 mM MgCl2 for 2 h at 37°C, and further incubated with 12 mM EDTA (Sigma) and 2 mM EGTA (Sigma) 10 min at 75°C. After that, the solution was treated with 200 µg/mL proteinase K (Sigma) in 0.5% SDS for 1 h at 45°C. Next, the viral DNA was precipitated incubating 1:1 vol of the sample with phenol:chloroform:isoamilic acid at 25:24:1. After centrifugation at 10,000 rpm for 3 min at RT, the watery fraction was transferred and further incubated with 0.1 volume of 3M acetic acid, pH 5.2; 1 µL LPA (Sigma), and 2 volumes of cold 100% ethanol for 1 h at −80°C. Then, the sample was centrifuged at 13,000 rpm for 30 min at 4°C, and the supernatant was discarded. The pellets were washed once with cold 70% ethanol and air-dried. Finally, the pellets were resuspended in 10 mM Tris-HCl, pH 8.8.

### Illumina sequencing and data analysis

Viral DNA was submitted to MicrobesNG (Birmingham, UK). Illumina libraries were prepared with NEBNext Ultra DNA Library Prep Kit (New England Biolabs). DNA sample was fragmented in a Covaris instrument and sequenced on an Illumina MiSeq device as paired-end (2 × 250 bp) reads. Illumina reads from each sequenced sample were trimmed using Trimmomatic v0.36 (81) and quality filtered (QF) with PrinSeq v1.2 (82).

Armc4-ΔCD2v-GFP genome was *de novo* assembled using Illumina reads, and Arm/07/CBM/c4 genome (LR881473) was used as a reference. Shortly, trimmed reads were assembled into contigs using SPAdes software [v 3.13.1; (83)]. Contigs were filtered against Arm/07/CBM/c4 reference genome using BLAST+ software [v 2.9.0; (84)]. Contigs with hits with the reference genome were extracted and extended using SSPACE software (85) and scaffolded into a single-genome sequence of 178,048 bp. Genome was annotated using Prokka.

Illumina reads of Arm/07/CBM/c4(Armc4)-CD2v-Nt and Arm/07/CBM/c4(Armc4)-CD2v-FL were aligned against Arm/07/CBM/c4(Armc4)-ΔCD2v-GFP genome using Bowtie2 software [v 2.3.5.1; (86)], and bam file was generated using Samtools (v 1.10) and visualized using IGV software (v 2.11.4). Variants were called using VarScan software (v 2.4.3) with a threshold of 0.4 for minimum variant frequency and a minimum coverage of 20 reads per base.

### RT-qPCR Assay

PAM were seeded in p60 plates (6 × 10^6^ cells/plate) and mock or infected with Arm07/CBM/c2 (WT) or NH/P68 (4 PFU/cell) in DMEM 10% porcine serum. At 8 or 16 hpi, the total RNA was extracted from cells using an RNeasy kit (Qiagen). cDNA was synthesized using a NZY first-strand cDNA synthesis kit (NZYTech). qPCR was performed using 12.5 ng of cDNA for each sample in a 384 plate and was run in CFX384 touch real-time PCR detection system (Bio-Rad) with SYBR green master mix (NZYTech). Gene expression levels were normalized to the housekeeping gene (18S rRNA), and these values were relative to the mock values. The primers used were 5′-GGCCCGAGGTTATCTAGAGTC-3′ and 5′-TCAAAACCAACCCGGTCA-3′ for porcine 18S rRNA detection and 5′-ACTTCCTAAGCCTTACAGTCGT-3′ and 5′-AGTGGTTGTGTTGAGGGACG-3′ for viral EP402R detection. Biological and experimental triplicates were used.

### Western blot assay

PAM or COS-1 cells were seeded in M6 plates (2–3 × 10^6^ cells/well) and treated as described in the figure’s legend. At the indicated times, the cells were collected, washed with PBS, and lysed with a radioimmunoprecipitation assay buffer (RIPA, Tris-HCl 50 mM, NaCl 150 mM, Triton 1%, deoxycholate 0.5%, SDS 0.1%, produced in-house) supplemented with protease and phosphatase inhibitors (Roche) for 30 min at 4 °C. Then, lysates were sonicated and centrifuged at 13,000 rpm for 10 min at 4 °C. The supernatants were collected, and equal amounts of protein were used. Samples were resolved by sodium dodecyl sulfate polyacrylamide gel electrophoresis (SDS-PAGE) and transferred to Immobilon-P membranes (Millipore, Burlington, MA, USA). The membranes were incubated with the following specific primary antibodies diluted in Tris-buffered saline (TBS) supplemented with 1% milk: anti-actin antibody (Sc-47778) from Santa Cruz Biotechnology (1/6,000); anti-myc-Tag antibody (9B11) from Cell Signaling #2276 (1/1,000); anti-p32 antibody (S1D8) for viral protein p32 (1/5,000) was produced in-house, anti-CD2v antibody for viral protein CD2v (1/10,000) was previously generated as described earlier (37), and anti-HA-Tag antibody (hibridome clone 12CA5) was generated in-house in CBMSO (1/1,000). Membranes were washed three times with TBS and exposed 1 h to specific peroxidase-conjugated secondary antibodies: anti-rabbit and anti-mouse immunoglobulin G coupled to peroxidase (1/5,000 and 1/2,000, respectively) from Amersham Biosciences and anti-mouse-IgGκ secondary antibody (1/1,000) from Santa Cruz Biotechnology. Chemiluminescence detection was performed using ECL Prime (Amersham).

### Identification of CD2v protein domains and N-glycosylation sites *in silico*

CD2v protein sequence was obtained from Arm/07/CBM/c2 genome by translating the genomic sequence of EP402R into amino acid sequence using Snapgene software. For the prediction of signal peptide of CD2v protein, SignalP software (Version 5.0) was used, with standard parameters. Transmembrane domains were predicted using TMHMM software (Version 2.0). Data gathered from SingalP and TMHMM software were confirmed using PSIPRED (Version 4.0) software, using the following options: PSIPRED for secondary structure prediction, MEMSAT for helix prediction, and Dompred for domain prediction. N-glycosylation sites were predicted using Net-N-Glyc software (Version 1.0).

### Comparison of amino acid sequences of signal peptides in different ASFV strains

Genomes for different ASFV strains were obtained from Genbank database, with the accession numbers that are specified in Table S4. The amino acid sequence for CD2v protein, corresponding to EP402R gene, was obtained directly from the genbank file downloaded for each genome. In those genomes where no annotation was provided, a sequence alignment using EP402R gene from Arm/07/CBM/c2 was performed using Snapgene software. The aligned region was analyzed for CDS, and the CDS corresponding to EP402R gene was translated into amino acid sequences using EMBOSS transeq software (87). Amino acid sequences were aligned using ClustalW software (87).

### Detection of surface CD2v in transfected COS-1 cells by fluorescence-activated cell sorting (FACS)

COS-1 cells were transfected with the indicated vectors with Metafectene (Biontex) as previously indicated (1 µg/10^6^ cells) and incubated for 6 h. After that, cells were infected with VV-T7 (MOI) = 0.5, as indicated, for further 16 h. Cells were then collected, washed in PBS, and incubated with Ghost Dye Red 780 (TONBO biosciences) (1 µg/mL) for 5 min at 37 °C in darkness. Then, the cells were centrifuged (2,000 rpm, 2 min) and washed with PSB-staining buffer (PBS 0.5% BSA) and stained with anti-myc (9B11, Mouse mAb #2276, Cell signaling) (1/100 diluted in PBS-staining buffer) for 30 min at 4 °C, followed by incubation with an anti-mouse Alexa Fluor-647 (diluted 1/500 in PBS-staining buffer) in the same conditions. Then, cells were fixed with 2% paraformaldehyde for 30 min at 4 °C and permeabilized with PBS-staining buffer 0.2% saponin for 15 min at RT. Finally, cells were stained with anti-myc (9B11, Mouse mAb #2276, Cell Signaling) (1/100 diluted in PBS-staining buffer 0.2% saponin) for 30 min at 4 °C, followed by incubation with an anti-mouse Alexa Fluor-488 (diluted 1/500 in PBS-staining buffer 0.2% saponin) in the same conditions. Finally, cells were analyzed in a FACSCanto A flow cytometer (BD Science) to determine the percentage of cells expressing CD2v in cell surface (647+) and/or expressing CD2v intracellular (488+). FACS analyses were performed in triplicate with biological duplicates. Error bars represent the standard deviation.

### CD2v digestion with endoglycosidases

About 10 × 10^6^ COS-1 cells were transfected with 1 µg/1 × 10^6^ cells of the specific vector using Metafectene transfection reagent (Biontex). At 6 hpt, cells were infected with VV-T7 (MOI = 0.5 PFU/mL). At 16 hpi, cells were collected and lysed with 1 mL of Pierce IP Lysis (ThermoFisher Scientific) and protease and phosphatase inhibitors for 2 h on ice. Lysates were centrifuged and precleared with Pierce Protein A/G Magnetic Beads (ThermoFisher Scientific). CD2v variants were immunoprecipitated using either Pierce Anti-c-Myc Magnetic Beads or anti-CD2v bonded to magnetic beads Pierce Protein A/G Magnetic Beads (ThermoFisher Scientific), as indicated, at 4 °C with rotation overnight. Different immunoprecipitates were eluted and non-treated or treated with either endoglycosidases PNGase-F or Endo-H (New England BioLabs). Briefly, IP elution was denaturalized using denaturalized buffer at 100 °C for 10 min and then chilled on ice, centrifuged at 11,000 rpm, and incubated with PNGase-F or Endo-H (1 h at 37 °C). Finally, samples were analyzed by Western blot.

## SUPPLEMENTAL MATERIAL

Supplemental tables and figure legends jvi.01030-23-s0001.docx Tables S1 to S4 and legends of Fig. S1 to S8.

Fig. S1 jvi.01030-23-s0002.tif Verification of mutations in Arm-ΔCD2v-GFP, Arm-CD2v-FL*, and Arm-CD2v-Nt by PCR.

Fig. S2 jvi.01030-23-s0003.tif Tunicamycin treatment prevents rosette formation in COS-1-infected cells with recombinant ASFV virus Arm-CD2v-Nt and Arm-CD2v-FL*.

Fig. S3 jvi.01030-23-s0004.tif Additional Western blot exposures.

Fig. S4 jvi.01030-23-s0005.tif CD2v-Ct domain is not glycosylated.

Fig. S5 jvi.01030-23-s0006.tif Sequence alignment of CD2v signal peptide sequences from ASFV strains of different genotypes.

Fig. S6 jvi.01030-23-s0007.tif Complementation of CD2v-Nt from NH/P68 with the signal peptide from E75, or exchange of sequences at the Nt end of CD2v-Nt from NH/P68 with CD2v sequence from E75/Ba71V, fails to recover the HAD phenotype.

Fig. S7 jvi.01030-23-s0008.tif Complementation of the CD2v-FL of NH/P68 with the “whole” CD2v signal peptides of both Arm/07 and Ba71V recovers HAD phenotype only when a Nt end sequence of CD2v from NH/P68 is deleted.

Fig. S8 jvi.01030-23-s0009.tif Complementation of the CD2v-FL of NH/P68 with the *in silico* predicted CD2v signal peptides of both Arm/07 and Ba71V fails to recover HAD phenotype even if the Nt end inhibitory sequence of CD2v from NH/P68 is deleted.

ASM does not own the copyrights to Supplemental Material that may be linked to, or accessed through, an article. The authors have granted ASM a non-exclusive, world-wide license to publish the Supplemental Material files. Please contact the corresponding author directly for reuse.
