# Supplementary material for: Signal peptide and N-glycosylation of N-terminal-CD2v determine the hemadsorption of African swine fever virus
Source: J Virol. 2023 Sep 28;97(10):e01030-23. doi: 10.1128/jvi.01030-23 (PMC10617588; doi:10.1128/jvi.01030-23)
Supplement: Supplemental tables and figure legends — Tables S1 to S4 and legends of Fig. S1 to S8. [file jvi.01030-23-s0001.docx]

**Supplementary material:**

**Table S1.** Variants of Armc4-CD2vNt or Armc4-CD2vFL* vs. Armc4 ΔCD2v-GFP identified by VarScan using a threshold of 40% of abundance.

| Variants of Armc4-CD2vNt vs. Armc4 ΔCD2v-GFP | | | | |
| --- | --- | --- | --- | --- |
| Position | Reference | Variant | Gene | Effect |
| 91408 | C | T | B602L | Asp235Asn |
| 122249 | C | T | NP1450L | Gly329Gly |
| Variants of Armc4-CD2vFL* vs. Armc4 ΔCD2v-GFP | | | | |
| Position | Reference | Variant | Gene | Effect |
| 91408 | C | T | B602L | Asp235Asn |
| 167277 | GA | G | MGF360-16R | Lys300Frameshift |

**Table S2.** Percentage of infected cells (COS-1 cells or PAM) showing rosettes after infection with different ASFV strains (for COS-1) or recombinant ASFV viruses (for PAMs). n= 600-1000.

|  | Cells | |  |
| --- | --- | --- | --- |
| ASFV strains | COS-1 | PAMs | **ASFV recombinant viruses** |
| E70 | 27.4 | 76.7 | **Arm-CD2v-FL*** |
| Arm/07 | 38.2 | 55.3 | **Arm-CD2v-Nt** |

**Table S3.** Percentage of transfected COS-1 cells with the indicated CD2v form or mutant showing rosettes after incubation with porcine erythrocytes. n= 500-1500.

| CD2v form or mutant | Transfected COS-1 cells |
| --- | --- |
| CD2v-FL_Ba71V | 25.0 |
| CD2v-FL_Arm | 28.1 |
| CD2v-Nt_Arm | 36.7 |
| CD2v-Nt_Arm_NQ1,3,4 | 11.8 |
| CD2v-Nt_Arm_NQ1,2 | 16.0 |
| CD2v-Nt_Arm_NQ4,6 | 17.9 |
| CD2v-Nt_Arm_NQ7,8 | 49.8 |
| CD2v-Nt_Arm_NQ1 | 46.6 |
| CD2v-Nt_Arm_NQ2 | 28.9 |
| CD2v-Nt_Arm_NQ3 | 31.8 |
| CD2v-FL_NH/P68ΔSeq+SP-Arm-_whole_ | 30.1 |
| CD2v-FL_NH/P68ΔSeq+SP-Ba71V-_whole_ | 30.1 |

**Table S4.** Features and accession number of ASFV strains used in the CD2v (EP402R) signal peptide sequence comparison.

| **Strain** | **Year** | **Genotype** | **Hemadsorbent** | **Accesion Number** |
| --- | --- | --- | --- | --- |
| NH/P68 | 1968 | I | No | NC_044943.1 |
| OURT88/3 | 1988 | I | No | NC_044957.1 |
| E75 | 1975 | I | Yes | NC_044958.1 |
| L60 | 1960 | I | Yes | NC_044941.1 |
| Liv13/33 | 1983 | I | Yes | MN318203.3 |
| SD/DY-I/21 | 2021 | I | No | MZ945537.1 |
| HeN/ZZ-P1/21 | 2021 | I | No | MZ945536.1 |
| Ba71V | 1971 | I | Yes | NC_001659.2 |
| Arm/07/CBM/c2 | 2020 | II | Yes | LR812933.1 |
| Paju/Pig/2019 | 2019 | II | Yes | MT748042.2 |
| Estonia2014 | 2014 | II | Yes^1^ | LS478113.1 |
| Tanzania/Rukwa/2017 | 2017 | II | Yes | LR813622.1 |
| Ind/AS/SD-02/2020 | 2020 | II | Yes | OL692743.1 |
| China/2018/AnhuiXCGQ | 2018 | II | Yes | MK128995.1 |
| DR80 | 1980 | I | Yes | ON185726.2 |
| Georgia 2007/1 | 2007 | II | Yes | FR682468.2 |
| Malawi Lil-20/1 | 1983 | VIII | ? | NC_044954.1 |
| RSA_2_2004 | 2004 | XX | ? | MN641877.2 |
| Ken06.Bus | 2006 | IX | ? | NC_044946.1 |
| RSA_2_2008 | 2008 | XXII | ? | MN336500.3 |
| Warthog | - | IV | ? | NC_044949.1 |
| Warmbaths | - | III | ? | NC_044950.1 |

**Figure S1. Verification of mutations in Arm-ΔCD2v-GFP, Arm-CD2v-FL* and Arm-CD2v-Nt by PCR.** PCR assay showing the presence/absence of either CD2v (1080 pb), CD2v-Nt (684pb) or GFP (1016 pb) genes in the Arm/07/CBM/c4 (WT), Arm-ΔCD2v-GFP, Arm-CD2v-FL* or Arm-CD2v-Nt genomes, using the oligos showed in Table 2.

**Figure S2. Tunicamycin treatment prevents rosette formation in COS-1-infected cells with recombinant ASFV virus Arm-CD2v-Nt and Arm-CD2v-FL*.** COS-1 cells were infected with Arm-ΔCD2v-GFP, Arm-CD2vFL* or Arm-CD2v-Nt (MOI= 1) in the presence or not of tunicamycin (5 µg/ml). After 16h, porcine erythrocytes were added to the medium and 24h later cells were observed under fluorescent/light microscope to identify rosettes. Arrows indicate rosettes. Scale bar 100µm.

**Figure S3.** **Additional Western blot exposures.** Additional Western blot exposures corresponding to Figure 6C (A), Figure 7A (B), Figure 8B (C) and Figure 10B (D).

**Figure S4. CD2v-Ct domain is not glycosylated.** COS-1 cells were transfected with expression vectors for CD2v-Ct, 6 hpt, cells were infected with VV-T7 (MOI= 0.5) in the presence or not of tunicamycine (5 µg/ml) for 16h. Samples were then lysed in RIPA buffer, separated by 10% SDS-PAGE, followed by immunoblotting with anti-CD2v and anti-actin antibodies.

**Figure S5. Sequence alignment of CD2v signal peptide sequences from ASFV strains of different genotypes.** Alignment of the amino acid sequences of Nt-CD2v obtained for different ASFV genotypes. Similar CD2v signal peptides are highlighted in colored boxes as follows: yellow for signal peptides similar to Arm/07/CBM/c2, red for signal peptides similar to E75, green for signal peptides similar to Ba71V. Inhibitory sequence found in CD2v of NH/P68 are highlighted in blue.

**Figure S6. Complementation of CD2v-Nt from NH/P68 with the signal peptide from E75, or exchange of sequences at the Nt end of CD2v-Nt from NH/P68 with CD2v sequence from E75/Ba71V, fails to recover the HAD phenotype.** (A) Schematic illustrating the sequence comparison of CD2v Nt-terminal sequences of Arm/07/CBM/c2, Ba71V, E75 or NH/P68 and signal peptides where appropriate. The yellow box highlights the in silico predicted signal peptide sequence for CD2v of Arm/07/CBM/c2, Ba71V and E75 (SP-Arm and SP-Ba71Vsp, SP-E75, respectively). In the green box, the predicted transmembrane sequence in CD2v of Ba71v (SP-Ba71V-tm) is highlighted. Finally, the blue box highlights the single sequence appearing at the Nt end of CD2v of NH/P68 that was substituted by the sequence highlighted in the purple box (Seq. inter). (B, C) COS-1 cells were transfected with empty vector or expression vectors for CD2v-Nt-Arm/07, CD2v-Nt-NH/P68, CD2v-Nt-NH/P68-Seq.inter, CD2v-Nt-NH/P68+SP-Ba71Vsp, CD2v-Nt-NH/P68+SP-Ba71Vtm or CD2v-Nt-NH/P68+SP-E75. 6 hpt, cells were infected with VV-T7 (MOI= 0.5) for 16h (B) lysed in RIPA buffer, separated by 10% SDS-PAGE, followed by immunoblotting with anti-myc, anti-CD2v and anti-actin antibodies, or (C) incubated with porcine erythrocytes for further 24h before observation under light microscope.

**Figure S7. Complementation of the CD2v-FL of NH/P68 with the “whole” CD2v signal peptides of both Arm/07 or Ba71V recovers HAD phenotype only when a Nt end sequence of CD2v from NH/P68 is deleted.** (A) Diagram showing the structure of CD2v-FL-NH/P68+ SP-Armwhole or SP-Ba71Vwhole and CD2v-FL-NH/P68ΔSeq+ SP-Armwhole or SP-Ba71Vwhole. (B, C) COS-1 cells were transfected with empty vector or expression vectors for CD2v-FL-Arm/07, CD2v-FL-NH/P68, CD2v-FL-NH/P68+SP-Armwhole, CD2v-FL-NH/P68+SP-Ba71Vwhole, CD2v-FL-NH/P68ΔSeq+ SP-Armwhole or CD2v-FL-NH/P68ΔSeq+ SP-Ba71Vwhole. 6 hpt, cells were infected with VV-T7 (MOI= 0.5) for 16h and (B) lysed in RIPA buffer, separated by 10% SDS-PAGE, followed by immunoblotting with anti-myc, anti-CD2v and anti-actin antibodies, or (C) incubated with porcine erythrocytes for further 24h before observation under light microscope. Arrows indicate rosettes (C).

**Figure S8. Complementation of the CD2v-FL of NH/P68 with the *in silico* predicted CD2v signal peptides of both Arm/07 or Ba71V fails to recover HAD phenotype even if the Nt end inhibitory sequence of CD2v from NH/P68 is deleted.** (A) Diagram showing the structure of CD2v-FL-NH/P68ΔSeq+SP-Arm, CD2v-FL-NH/P68ΔSeq+SP-Ba71Vsp or CD2v-FL-NH/P68ΔSeq+SP-Ba71Vtm. (B, C) COS-1 cells were transfected with empty vector or expression vectors for CD2v-FL-Arm/07, CD2v-FL-NH/P68, CD2v-FL-NH/P68ΔSeq+SP-Arm, CD2v-FL-NH/P68ΔSeq+SP-Ba71V-sp or CD2v-FL-NH/P68ΔSeq+SP-Ba71V-tm. 6 hpt, cells were infected with VV-T7 (MOI= 0.5) for 16h (B) lysed in RIPA buffer, separated by 10% SDS-PAGE, followed by immunoblotting with anti-myc, anti-CD2v and anti-actin antibodies, or (C) incubated with porcine erythrocytes for further 24h before observation under light microscope.
