# Supplementary figures and images for: Signal peptide and N-glycosylation of N-terminal-CD2v determine the hemadsorption of African swine fever virus
Source: J Virol. 2023 Sep 28;97(10):e01030-23. doi: 10.1128/jvi.01030-23 (PMC10617588; doi:10.1128/jvi.01030-23)

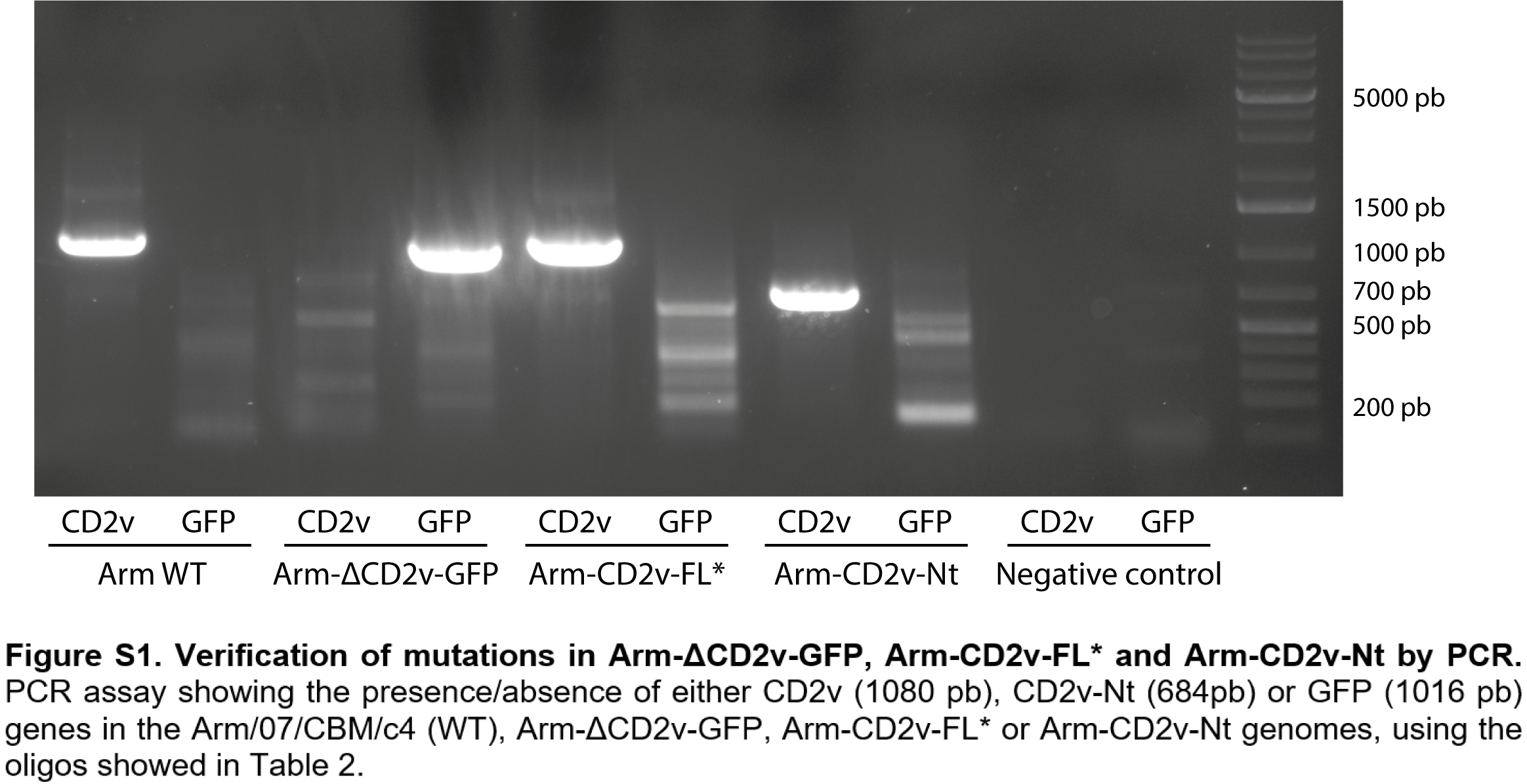

Supplement: Fig. S1 — Verification of mutations in Arm-ΔCD2v-GFP, Arm-CD2v-FL*, and Arm-CD2v-Nt by PCR. [file jvi.01030-23-s0002.tif]

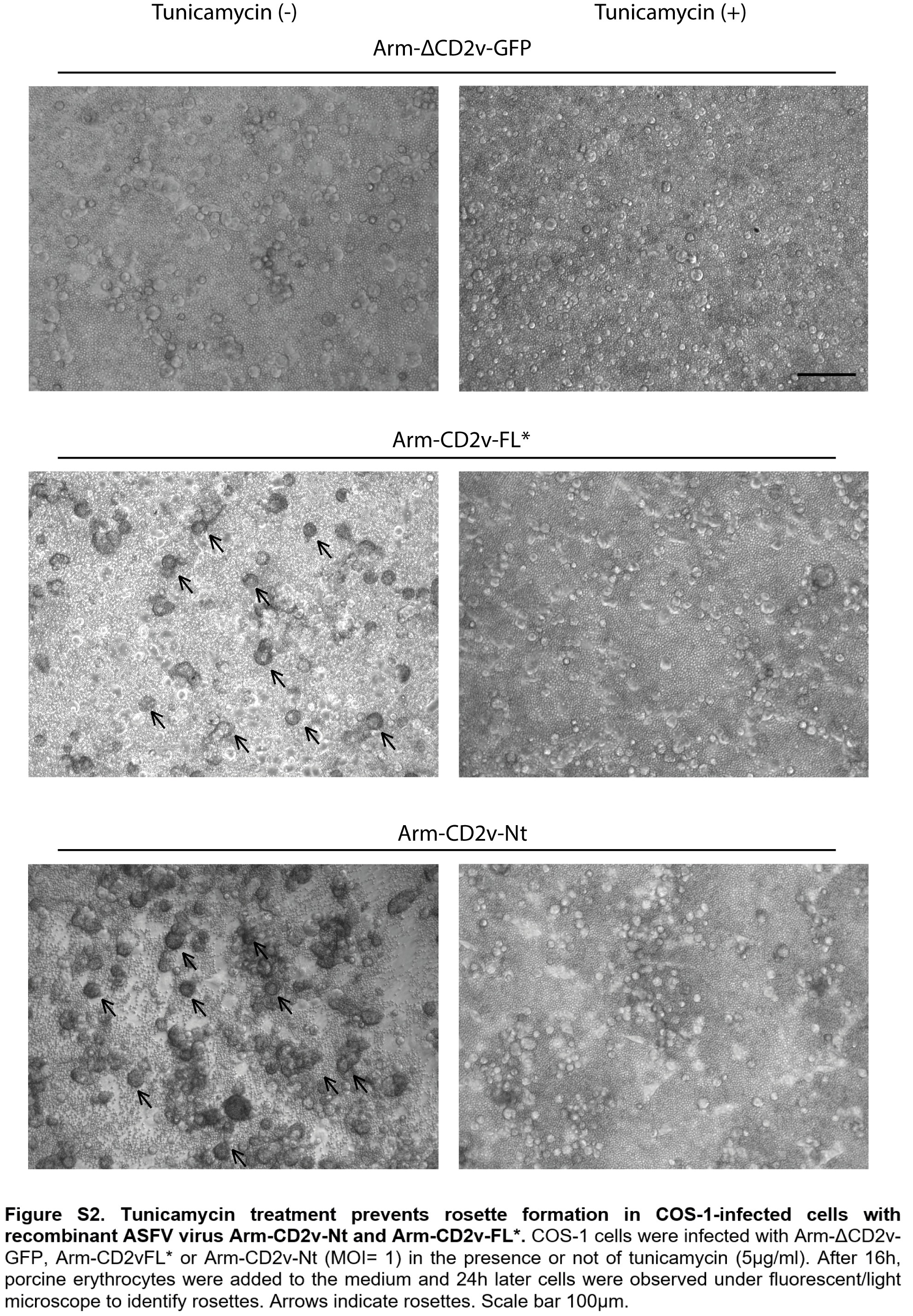

Supplement: Fig. S2 — Tunicamycin treatment prevents rosette formation in COS-1-infected cells with recombinant ASFV virus Arm-CD2v-Nt and Arm-CD2v-FL*. [file jvi.01030-23-s0003.tif]

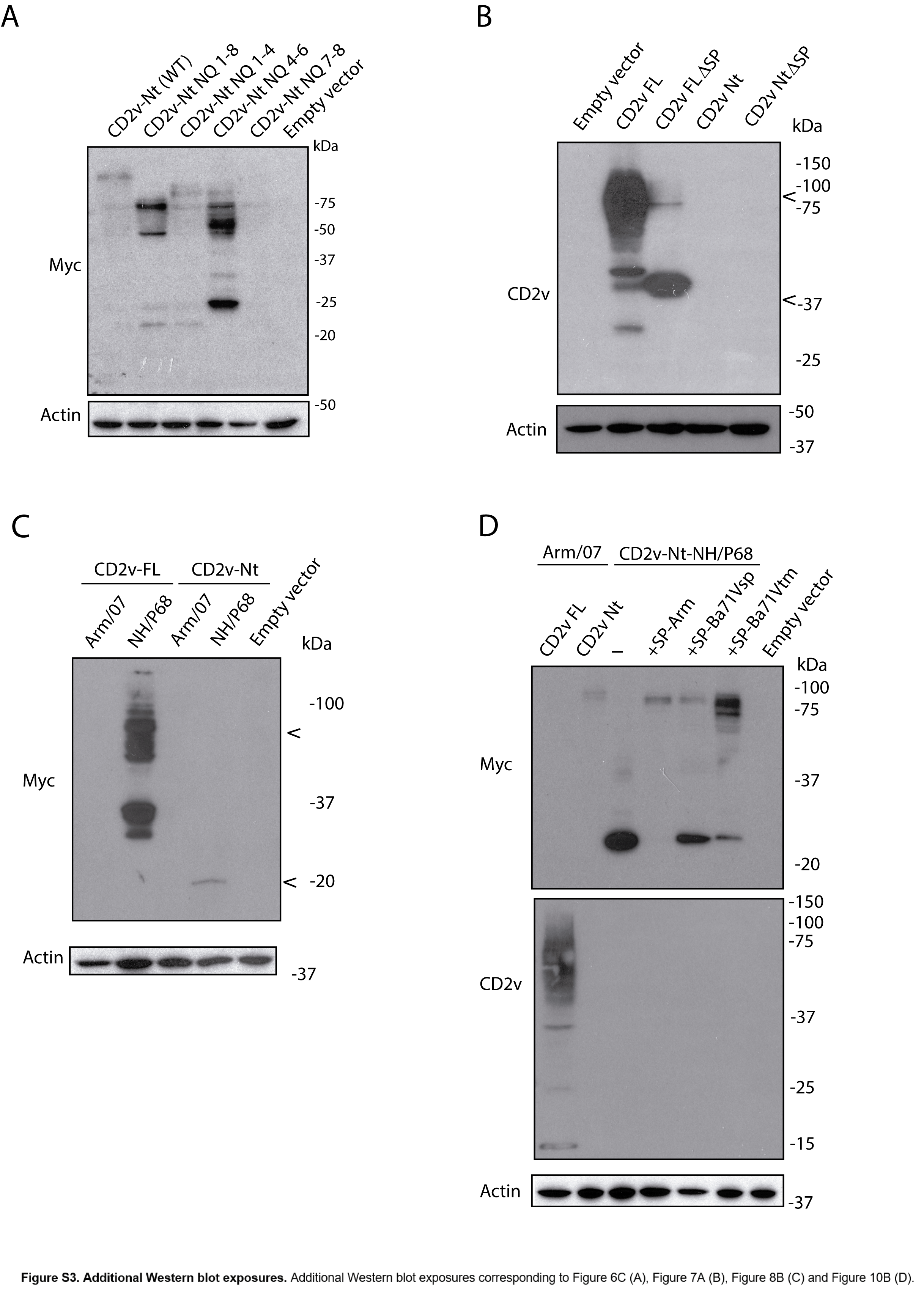

Supplement: Fig. S3 — Additional Western blot exposures. [file jvi.01030-23-s0004.tif]

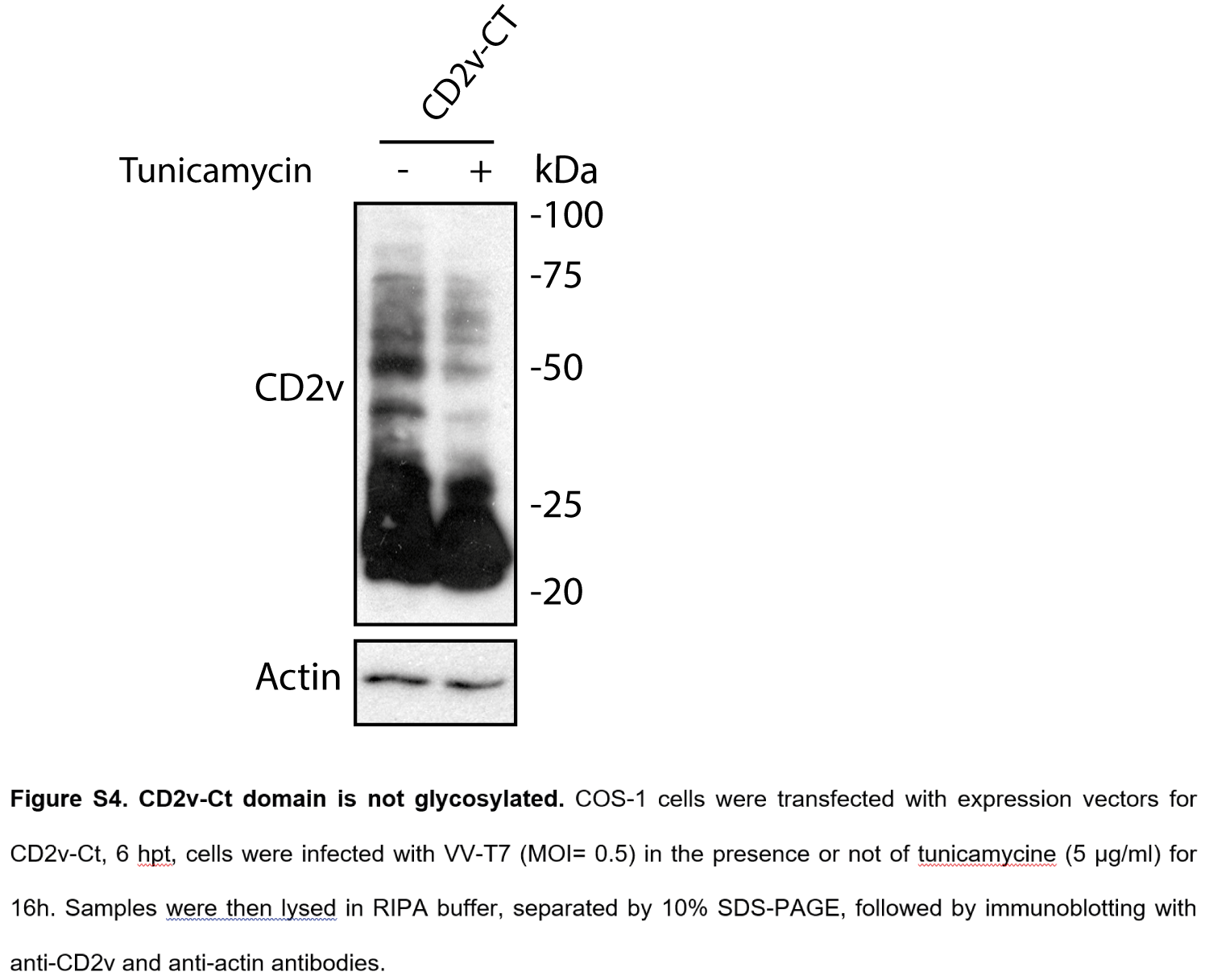

Supplement: Fig. S4 — CD2v-Ct domain is not glycosylated. [file jvi.01030-23-s0005.tif]

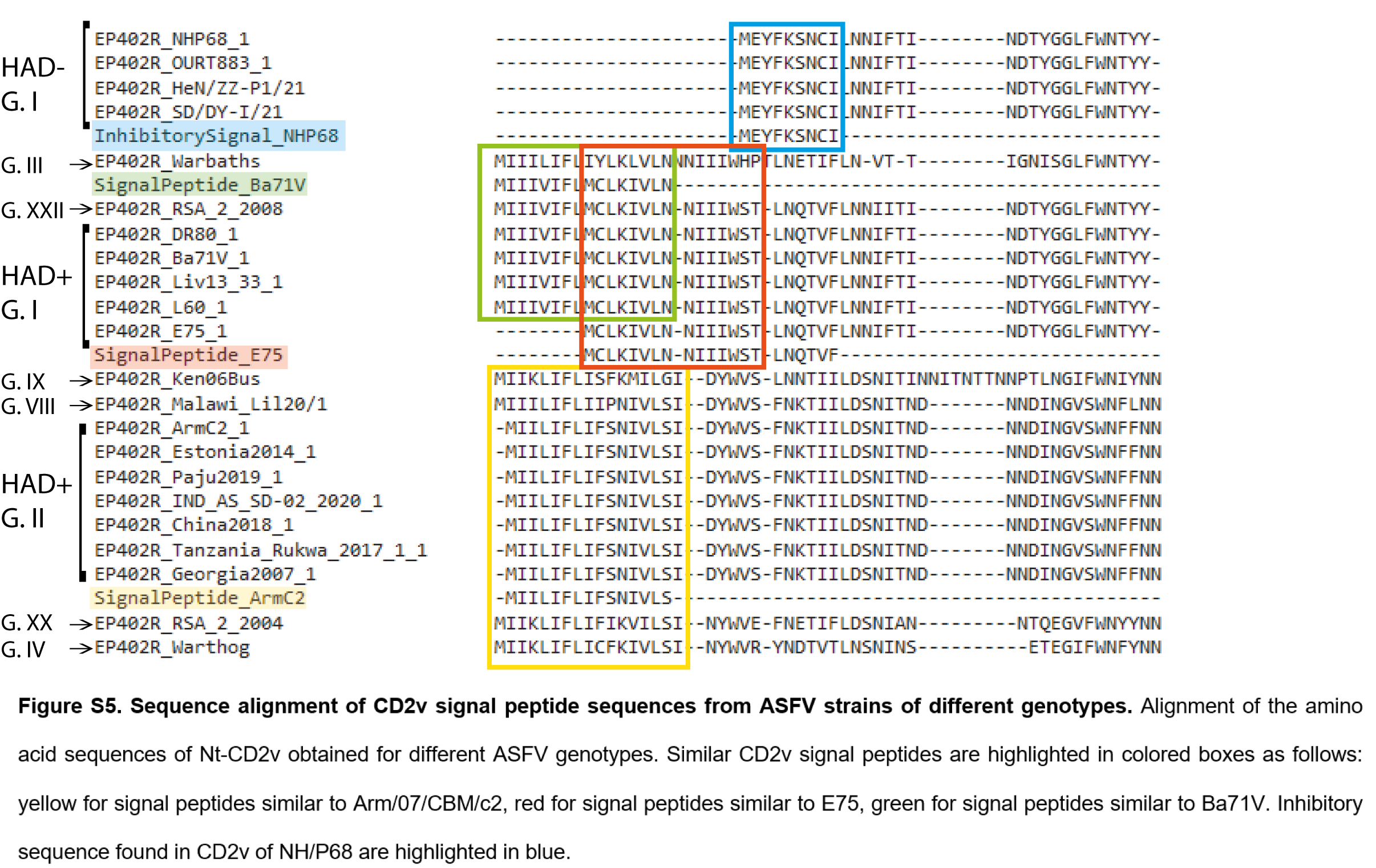

Supplement: Fig. S5 — Sequence alignment of CD2v signal peptide sequences from ASFV strains of different genotypes. [file jvi.01030-23-s0006.tif]

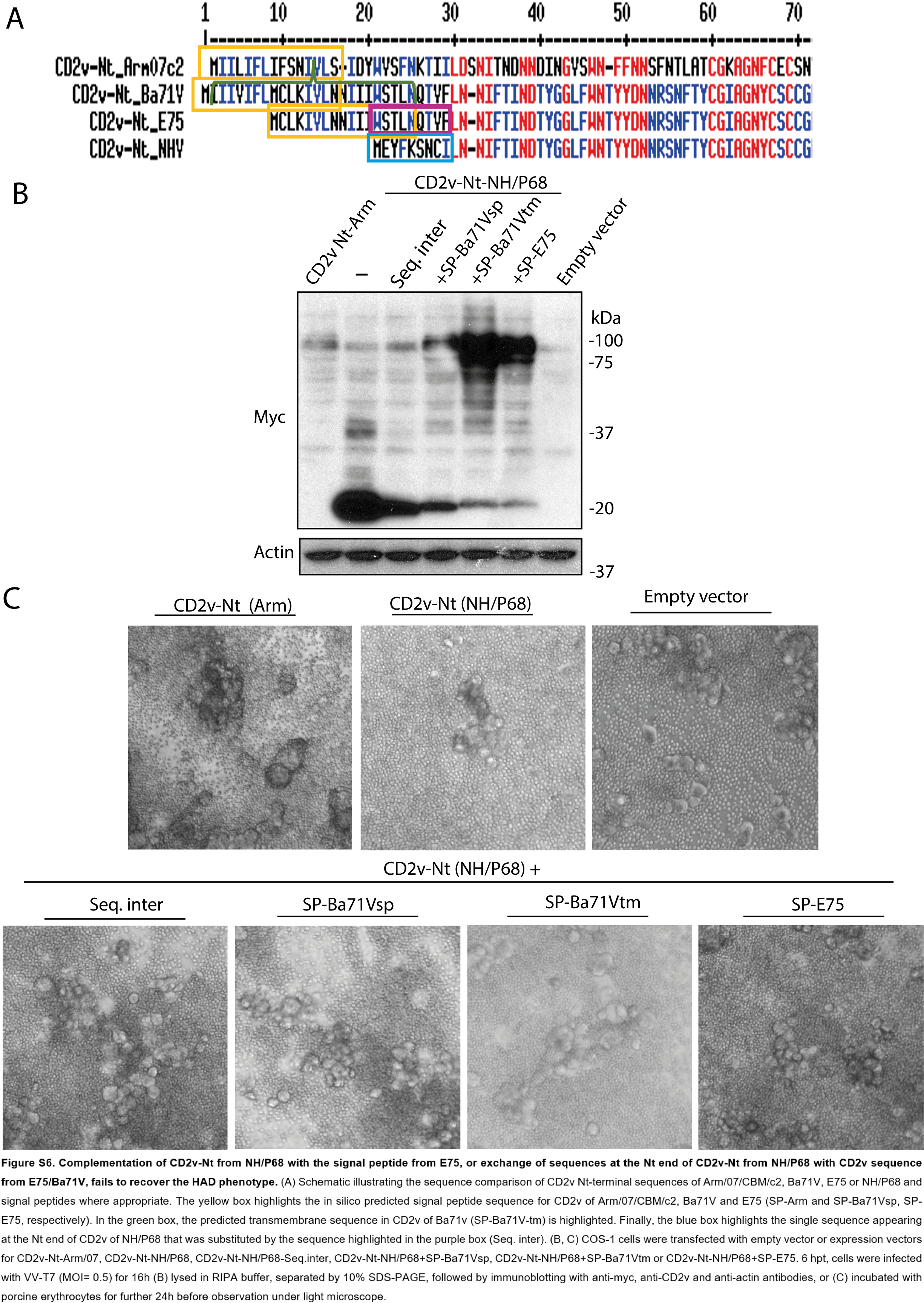

Supplement: Fig. S6 — Complementation of CD2v-Nt from NH/P68 with the signal peptide from E75, or exchange of sequences at the Nt end of CD2v-Nt from NH/P68 with CD2v sequence from E75/Ba71V, fails to recover the HAD phenotype. [file jvi.01030-23-s0007.tif]

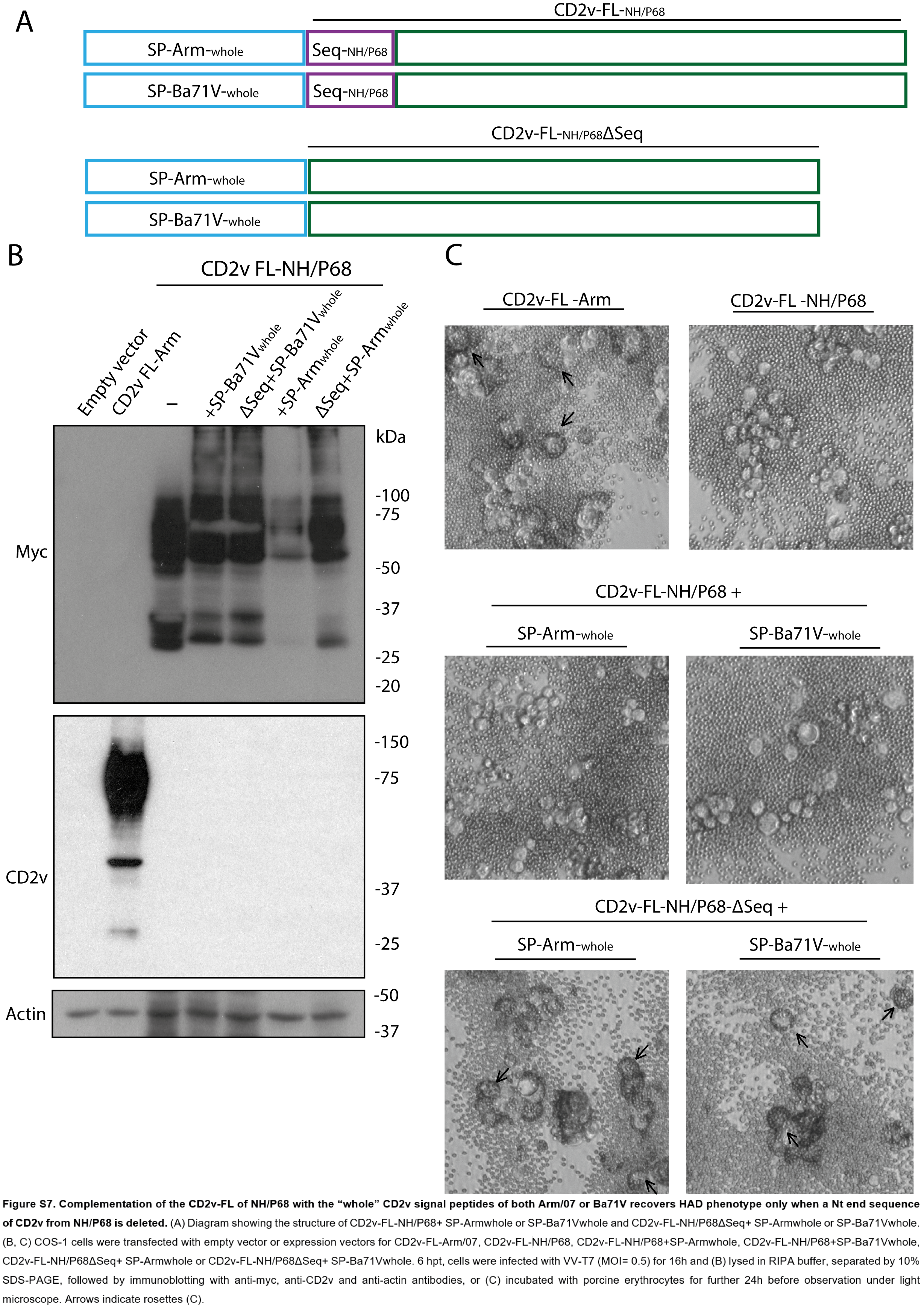

Supplement: Fig. S7 — Complementation of the CD2v-FL of NH/P68 with the “whole” CD2v signal peptides of both Arm/07 and Ba71V recovers HAD phenotype only when a Nt end sequence of CD2v from NH/P68 is deleted. [file jvi.01030-23-s0008.tif]

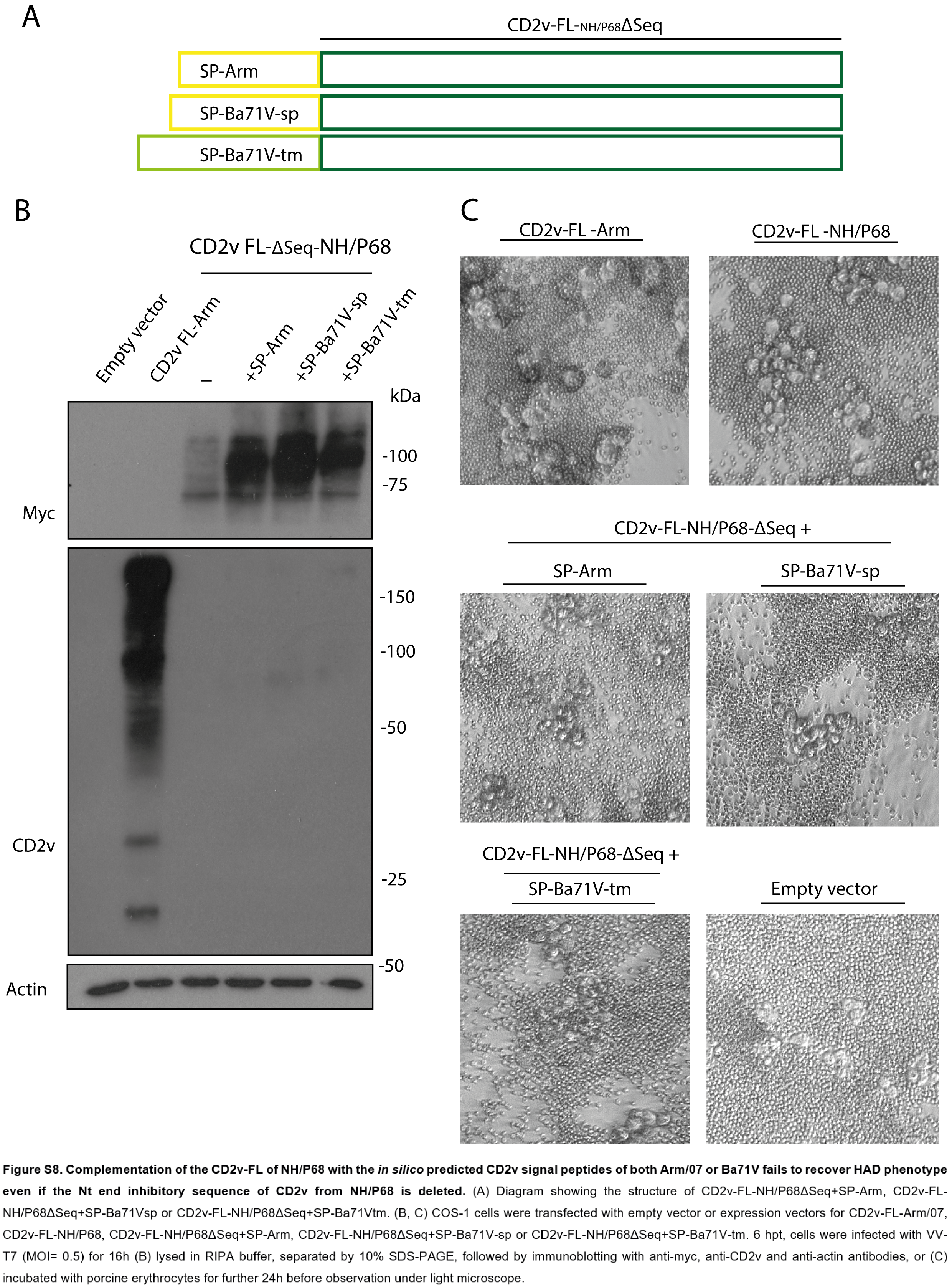

Supplement: Fig. S8 — Complementation of the CD2v-FL of NH/P68 with the in silico predicted CD2v signal peptides of both Arm/07 and Ba71V fails to recover HAD phenotype even if the Nt end inhibitory sequence of CD2v from NH/P68 is deleted. [file jvi.01030-23-s0009.tif]
